# Heterogeneous multiple soft millirobots in three-dimensional lumens

**DOI:** 10.1126/sciadv.adq1951

**Published:** 2024-11-06

**Authors:** Chunxiang Wang, Tianlu Wang, Mingtong Li, Rongjing Zhang, Halim Ugurlu, Metin Sitti

**Affiliations:** ^1^ Physical Intelligence Department, Max Planck Institute for Intelligent Systems, 70569 Stuttgart, Germany.; ^2^ Department of Information Technology and Electrical Engineering, ETH Zürich, 8092 Zürich, Switzerland.; ^3^ Department of Mechanical Engineering, University of Hawaiʻi at Mānoa, Honolulu, HI 96822, USA.; ^4^ Zentrum für Radiologie Heilbronn, 74177 Heilbronn, Germany.; ^5^ School of Medicine and College of Engineering, Koç University, 34450 Istanbul, Turkey.

## Abstract

Miniature soft robots offer opportunities for safe and physically adaptive medical interventions in hard-to-reach regions. Deploying multiple robots could further enhance the efficacy and multifunctionality of these operations. However, multirobot deployment in physiologically relevant three-dimensional (3D) tubular structures is limited by the lack of effective mechanisms for independent control of miniature magnetic soft robots. This work presents a framework leveraging the shape-adaptive robotic design and heterogeneous resistance from robot-lumen interactions to enable magnetic multirobot control. We first compute influence and actuation regions to quantify robot movement. Subsequently, a path planning algorithm generates the trajectory of a permanent magnet for multirobot navigation in 3D lumens. Last, robots are controlled individually in multilayer lumen networks under medical imaging. Demonstrations of multilocation cargo delivery and flow diversion manifest their potential to enhance biomedical functions. This framework offers a solution to multirobot actuation benefiting applications across different miniature robotic devices in complex environments.

## Teaser

Leveraging heterogeneous resistance, multiple wireless soft millirobots are deployed in 3D lumens for biomedical applications.

## INTRODUCTION

Wireless miniature robots have the potential to revolutionize biomedical engineering with their shape-adaptable and minimally invasive access and navigation in enclosed, tortuous, and unstructured spaces that are clinically inaccessible or risky for tethered tools (*1*–*3*). These small-scale devices can be actuated by external fields, such as light (*4*, *5*), ultrasound (*6*–*8*), and magnetic field (*9*, *10*) for various medical purposes. Among actuation methods, magnetically driven shape-programmable soft robots are particularly promising deep inside the human body (*11*–*13*) for various medical applications, such as targeted drug or cell delivery (*14*–*16*), vascular embolization (*17*–*19*), hyperthermia (*20*), and physiological property sensing (*21*–*23*). Such robots have the advantage of being harmless (*11*, *24*), agile (*9*, *25*), shape-adaptable (*10*, *26*, *27*), and easy to miniaturize down to milli- and micrometer scale (*11*, *12*). Deploying multiple of these magnetic soft robots can further enhance the efficacy of biomedical functions spatiotemporally. Multiple robots could concurrently execute therapeutic and diagnostic procedures at diverse locations. Examples include the interventional embolization of multiple cerebral tumors (*28*–*32*) and aneurysms (*33*–*35*), as well as the distributed sensing of physiological properties facilitated by integrated electronic modules (*36*–*39*). In addition, the efficiency of therapeutic interventions can be notably boosted, enabling a prompter response to acute diseases, such as thrombolysis for ischemic stroke (*40*–*42*) at multiple locations. In contrast, the dependence on a single robot navigating to the designated location, applying the medical functions, returning to the start location, and repeating the procedures for different locations would notably prolong the operation duration and miss the ideal treatment time window.

So far, deploying and controlling multiple magnetic miniature robotic devices in three-dimensional (3D) lumens under physiologically relevant conditions have not been achieved yet. Challenges include the global influence of the external magnetic fields on all agents with magnetic materials and the poor understanding of interactive environmental factors, such as surface contact and fluid flow. Current strategies for controlling multiple magnetic robots can be classified into four categories. One method involves generating localized force to trap robots (*43*–*45*) or program their collective formation (*46*–*48*) with a specialized substrate or device, while another strategy uses specialized boundaries to mechanically anchor individual robots (*49*, *50*). Their limited 2D substrate or lateral boundary hinders realistic medical applications in 3D space. An alternative approach leverages differences in magnetic or geometrical properties among robots to induce varied responses to the same control input, which can be used to plan their heterogeneous dynamics or kinematics with global actuation signals (*51*–*55*). Furthermore, strategic programming of the control signal of the electromagnetic coils or placing/orienting a permanent magnet (PM) can generate spatially selective magnetic fields or gradients to independently control two identical or nonidentical robots (*56*–*59*). These two methods rely on the precise fabrication and modeling of the robots, and the arrangement of actuation signals usually involves solving a nonconvex optimization problem (*56*) and the complicated coordination of multiple magnetic actuation setups (*60*), limiting the number of controllable robots. In addition, they are typically demonstrated in well-controlled, static environments rather than dynamic physiological conditions with pulsatile flow.

**Fig. 1.**
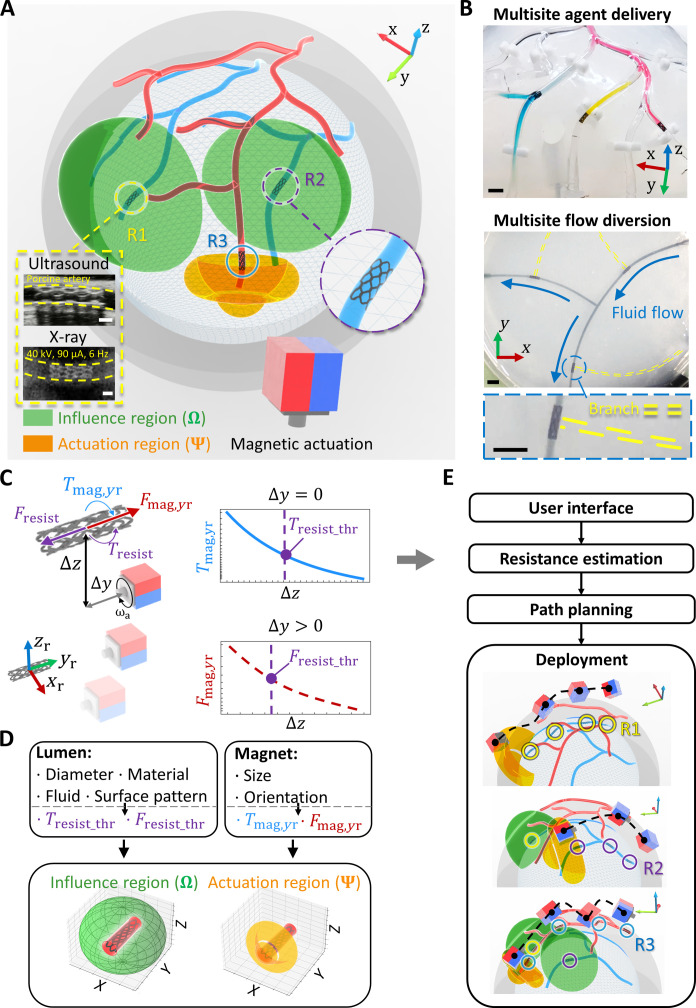
Overall concept of deploying multiple magnetic soft robots in 3D lumens. (**A**) Independent control of multiple stent-shaped robots facilitated by the influence region (Ω; shown by green) and actuation region (Ψ; shown by orange). By placing the actuation magnet outside Ω of the already deployed robots R1 and R2 while inside Ψ of the target robot to be actuated (R3), the individual robot deployment without affecting already deployed robots can be achieved. The subfigures illustrate the stent-shaped robot under medical imaging, where the yellow dotted lines denote lumens. Scale bars, 1 mm. (**B**) Demonstration of concurrent multisite cargo delivery and flow diversion. The yellow dotted lines illustrate the branch lumens. Scale bars, 5 mm. (**C**) Online estimation of the local resistance leveraged for independent robot control. The resistance is quantified using the magnetic torque and force at the critical point, observed by the initiation of robot rotation and translation, which is further used to calculate Ω and Ψ. (**D**) Factors influencing Ψ and Ω. For a given robot design, the size and shape of resistance Ω and Ψ are primarily associated with the material, stiffness, diameter, fluid flow, surface pattern of lumens, and the size and orientation of the N45 magnet. (**E**) Schematic of the overall deployment process using the estimated resistance and computed Ω and Ψ. The initial estimation of robot resistance along the designated lumen informs the path-planning process to generate the robot deployment order and magnet paths for multirobot deployment.

To tackle the fundamental challenges of magnetic miniature multirobot control toward medical applications, we propose a framework leveraging the shape-adaptive robotic design and heterogeneity in resistance stemming from friction and fluid drag, which can work in complex 3D tubular structures with pulsatile fluid flow inside, e.g., blood vessels. Actuated by a translating and rotating PM on a 7–degrees-of-freedom (DOF) robotic arm, the stent-structured shape-adaptive soft robots sequentially navigate through a multilayer 3D tubular network to the designated locations, as illustrated in Fig. 1A. Using the robot deformation–induced variation in resistance, the independent robot control is achieved by strategically placing PM outside the influence region (Ω) of the already deployed robots R1 and R2 while inside the actuation region (Ψ) of the target robot R3. This strategy ensures that R1 and R2 are constrained from rotation due to insufficient magnetic torques while enabling R3 to advance with strong magnetic torque and force. To the best of our knowledge, for the first time, a multirobot system capable of independently controlling a substantial number of magnetic soft robots (over five) in 3D lumens with internal fluidic flow at the millimeter scale is presented. Through concurrent multilocation cargo delivery and flow diversion (Fig. 1B) demonstrations, our proposed system is shown to have the potential to open avenues for a wide range of biomedical applications by deploying a group of soft robots equipped with diverse functional modules to hard-to-reach areas deep inside the human body for targeted therapeutic agent delivery, vascular embolization, physiological property sensing, hyperthermia, and other medical functions at multisite lesions.

## RESULTS

### Method of controlling multiple magnetic soft robots

The multirobot deployment framework is based on three key components: resistance-based influence region (Ω) and actuation region (Ψ) for independent robot control, variations of local resistance when interacting with surrounding lumens, and path planning for navigating multiple robots through the 3D lumen environment. A stent-structured magnetic soft robot is adopted for demonstrating the framework, with the retrievably adaptive locomotion in tortuous 3D lumens and the self-anchoring capability to withstand the fluid flow (*10*). PM is chosen for magnetic actuation to generate a strong magnetic field and field gradient given its advantages in compactness, easy extension to human scale, mobility when attached to a robotic arm, and heat-free operations compared with electromagnetic actuation (*24*, *61*). The challenge of using PM persists in the complex nature of the generated magnetic fields compared with the straightforward magnetic field and gradient control using electromagnetic coils (*24*).

Independent robot control is achieved via the interplay between magnetic actuation and local resistance stemming from friction and fluid drag. Using the rotational and translational magnetic actuation, the magnetic torque around the robot-body-attached *y*_r_ axis, *T*_mag,*y*r_, overcomes the resistive torque *T*_resist_ for robot rotation, followed by the magnetic force along the *y*_r_ axis, *F*_mag,*y*r_, surpassing the resistive force *F*_resist_ to propel the robot forward. To initiate robot rotation and translation, *T*_mag,*y*r_ and *F*_mag,*y*r_ should exceed the resistive torque threshold *T*_resist_thr_ and the resistive force threshold *F*_resist_thr_, respectively. With a PM approaching the robot, magnitudes of *T*_resist_thr_ and *F*_resist_thr_ are estimated by the corresponding *T*_mag,*y*r_ and *F*_mag,*y*r_ at the critical magnet positions where the robot starts rotating and translating, respectively, as shown in Fig. 1C. The estimated *T*_resist_thr_ and *F*_resist_thr_ are subsequently used to compute the robot-centered Ω and Ψ, respectively. Placing the PM outside Ω inhibits robot rotation, as *T*_mag,*y*r_ is less than *T*_resist_thr_. In contrast, robot rotation and translation are enabled by positioning the PM inside its Ψ, where *T*_mag,*y*r_ and *F*_mag,*y*r_ exceed *T*_resist_thr_ and *F*_resist_thr_, respectively. Leveraging the interplay between magnetic actuation and local resistance, independent control of the robot is achieved by strategically positioning the PM within the Ψ of the desired robot while avoiding the Ω of other robots.

The size and shape of Ω and Ψ can be dynamically regulated by actuating the robot to different locations of the lumen with various diameters or adjusting the PM size (*s*_mag_) and orientation, as illustrated in Fig. 1D. The lumen diameter, material, stiffness, surface pattern, and fluid flow affect *T*_resist_thr_ and *F*_resist_thr_, which further influences the size of Ω and Ψ. Increased lumen surface roughness or reduced ϕ_lumen_ lead to a larger *T*_resist_thr_ and consequently a smaller Ω, due to a larger friction coefficient or normal force, respectively. Moreover, the PM orientation and *s*_mag_ affect the spatial distribution and magnitude of *T*_mag,*y*r_ and *F*_mag,*y*r_, which further affects the shape and size of Ω and Ψ, respectively. Using the estimated *T*_resist_thr_ and *F*_resist_thr_ and computed Ω and Ψ, the overall deployment process is illustrated in Fig. 1E. Starting with the user-specified inputs through the interface, including the 3D lumen shape, lumen diameters, and designated robot destinations, the estimated resistance informs the path planning algorithm to further generate the PM paths to sequentially navigate each robot through the complex tubular network at a specific order, ensuring that the PM is consistently outside the Ω of deployed robots while maximizing the magnetic force for the actuated robot. Executing the preplanned magnet paths and the deployment order, a 6-DOF robotic arm (fig. S1) is used for supervised autonomous navigation of multiple robots to user-assigned locations in 3D lumens with pulsatile flow, monitored by the real-time optical or medical imaging, such as ultrasound and x-ray imaging (Fig. 1A).

### Resistance estimation and determination of two regions

To investigate the independent robot control via a PM, we analyze the magnetic actuation. The generated magnetic field by a PM rotates around a constant axis (*62*), and the magnitude of the rotating magnetic field projected onto the *x*_r_−*z*_r_ plane of the robot-body-attached coordinate, **B***_xz_*, is an ellipse with the maximum and minimum magnetic fields **B**_max,*xz*_ and **B**_min,*xz*_ (fig. S2), which induces *T*_mag,*y*r_ along the *y*_r_ axis for robot rotation. The robot is free of the magnet influence when the maximum magnetic torque (*T*_max_ = ∣**B**_max,*xz*_‖**m**_r_∣) is beneath *T*_resist_thr_, where the robot is kept from rotation due to the insufficient *T*_mag,*y*r_ to overcome *T*_resist_thr_. The magnetic moment of the robot is denoted by **m**_r_. In contrast, to enable the continuous rotation of the robot, the minimum magnetic torque (*T*_min_ = ∣**B**_min,*xz*_‖**m**_r_∣) should surpass *T*_resist_thr_ to ensure that *T*_mag,*y*r_ is constantly larger than *T*_resist_thr_. Under the continuous rotation, *F*_mag,*y*r_ along the *y*_r_ axis tries to pull the robot forward, fluctuating periodically with the rotating magnetic field on the *x*_r_−*z*_r_ plane, as illustrated in fig. S2. The prerequisite for robot translation is formulated as the mean magnetic force {*F*_mean_ = mean[*F*_mag,*y*r_(φ_a_)] for φ_a_ ∈ [0°,360°]} surpassing *F*_resist_thr_, where φ_a_ is the rotation angle of the PM. To actuate the desired robot, *T*_min_ and *F*_mean_ should exceed *T*_resist_thr_ and *F*_resist_thr_, respectively. The detailed analysis of *T*_max_, *T*_min_, and *F*_mean_ can be seen in the “Force modeling and analysis” section in Materials and Methods.

**Fig. 2.**
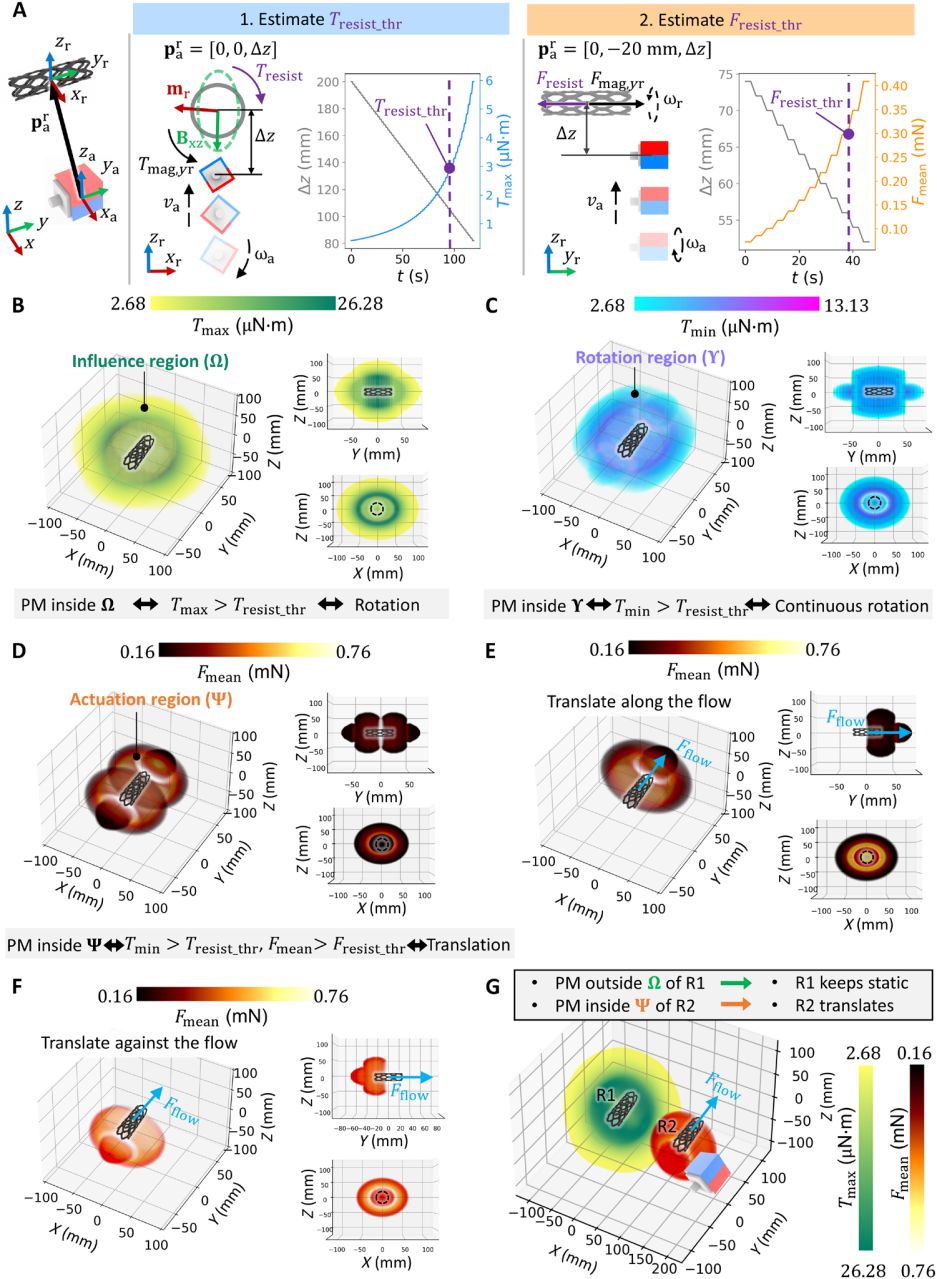
Resistance estimation and determination of the influence and actuation regions. (**A**) Resistance estimation procedure. The resistive torque threshold *T*_resist_thr_ is estimated from the maximum magnetic torque *T*_max_ at the initiation of robot rotation, while the resistive force threshold *F*_resist_thr_ is calculated with the mean magnetic force *F*_mean_ when the robot starts moving. The dotted green ellipsis denotes the magnitude of the magnetic field projected on the *x*_r_−*z*_r_ plane during actuator-magnet rotation. The frequency of the rotating PM is 1 Hz. (**B** and **C**) Quantification of the influence region Ω and rotation region Υ. When the PM is outside, the robot keeps static as *T*_max_ is beneath *T*_resist_thr_. When the PM is inside Ω while outside Υ, the robot rotates discontinuously. When inside Υ, the minimum magnetic torque *T*_min_ surpasses *T*_resist_thr_, enabling continuous rotation of the robot. (**D**) Quantification of the actuation region Ψ. Ψ lies within Υ, where *T*_min_ > *T*_resist_thr_ and *F*_mean_ > *F*_resist_thr_, facilitating continuous rotation and translation of the robot. (**E** and **F**) Variation in Ψ shapes with fluidic flow. Ψ shrinks as the robot moves against the flow, due to a larger *F*_resist_thr_ and a consequent required closer proximity of the PM to the robot. In contrast, Ψ expands as the robot locomotes along the flow. (**G**) Mechanism for multirobot control. Independent robot control is achieved by strategically placing the PM inside Ψ of the target robot (R2) while outside Ω of the already deployed robot (R1). In all figures, the 50-mm cubic PM is parallel to robots.

We propose a method to estimate the resistance properties (*T*_resist_thr_ and *F*_resist_thr_) online with a PM approaching the robot, as shown in Fig. 2A. First, a 50-mm PM is placed at $p_{a}^{r}={\left[0,0,l_{\text{min}}\right]}^{T}$ away from the robot to make **m**_r_ align with the *x*_r_ axis, during which the PM magnetic moment (**m**_a_) is fixed along the *x*_a_ axis. *l*_min_ is the minimal distance between the robot and PM to avoid collision between the PM and the surrounding environment. Subsequently, the PM relocates to $p_{a}^{r}={\left[0,0,\Delta z\right]}^{T}$ and then approaches the robot stepwise. The initial Δ*z* should be sufficiently large to disable the robot rotation due to a small *T*_mag,*y*r_. At the rotation critical point ($p_{a}^{r}={\left[0,0,{\Delta z}_{\text{critical}\_r}\right]}^{T}$), the discontinuous rotation of the robot is initiated with *T*_max_ surpassing *T*_resist_thr_, and *T*_max_ at $p_{a}^{r}={\left[0,0,{\Delta z}_{\text{critical}\_r}+1\;\text{mm}\right]}^{T}$ is formulated as *T*_resist_thr_. Furthermore, the PM advances to $p_{a}^{r}={\left[0,-20\;\text{mm},\Delta z\right]}^{T}$, and Δ*z* further decreases, during which the robot rotates continuously with *T*_min_ larger than *T*_resist_thr_. When *F*_mean_ exceeds *F*_resist_thr_ at the translation critical point $p_{a}^{r}={\left[0,-20\;\text{mm},{\Delta z}_{\text{critical}\_t}\right]}^{T}$, the robot moves forward, through which *F*_resist_thr_ is determined.

On the basis of the estimated *T*_resist_thr_ and *F*_resist_thr_, we introduce three robot-centered 3D regions to quantify the robot behavior under the PM actuation. First, the influence region (Ω) and rotation region (Υ) describe the robot rotation quantitatively, as illustrated in Fig. 2 (B and C) and fig. S3. The robot rotates discontinuously or continuously when the PM is inside Ω, where *T*_max_ is larger than *T*_resist_thr_. Furthermore, placing the PM inside Υ leads to the continuous rotation of the robot, where *T*_min_ is larger than *T*_resist_thr_. Notably, because of the serious misalignment between the robot and PM rotation axes, the robot cannot rotate continuously even if the PM is quite close to it in the corner region inside Ω while outside Υ (figs. S2 and S3). Within Υ, *F*_mean_ is calculated at each point, based on which the actuation region (Ψ) is obtained by excluding those points whose *F*_mean_ is smaller than *F*_resist_thr_ (Fig. 2D and fig. S4). Since the robot locomotes inside a fluid-filled lumen, *F*_resist_thr_ depends on the flow drag force *F*_flow_, which leads to a smaller Ψ against the fluid flow (Fig. 2E) and a larger Ψ along the flow (Fig. 2F). The shape and size of these three regions are determined by the magnitudes of local resistance *T*_resist_thr_ and *F*_resist_thr_, the magnitude of **m**_a_, and the direction of the PM rotation axis *y*_a_. As shown in fig. S5, these regions shrink under a smaller ∣**m**_a_∣ due to the decreased *T*_mag,*y*r_ and *F*_mag,*y*r_ from the PM, while the shapes of these regions are distorted due to the asymmetric distribution of *T*_mag,*y*r_ and *F*_mag,*y*r_ when the *y*_a_ axis misaligns with the *y*_r_ axis (fig. S6). The experimental validation of Ω, Υ, and Ψ is demonstrated in figs. S7 to S9.

To achieve independent robot control, the PM should be inside Ψ of the actuated robot with the *y*_a_ axis aligned parallel to the *y*_r_ axis, while remaining outside Ω of deployed robots (Fig. 2G). The influence of the PM orientation on decoupled control is investigated in fig. S10, with the key finding that altering the PM heading angle ψ around the *y*_a_ axis or elevation angle θ around the *x*_a_ axis does not notably contribute to the deployment efficiency. The evaluation metric used for quantifying the effect of decoupled control is the Intersection over rotation region (*IoR*), defined as the intersection area of Ω and Υ over the area of Υ. A smaller *IoR* corresponds to a reduced intersection, indicative of a more effective decoupled control. In a scenario involving two parallel robots, increasing ψ or θ of the PM leads to a relatively smaller *IoR*, but *F*_mean_ experiences a drastic reduction for the actuated robot, with *IoR* decreasing by 12% while *F*_mean_ falling by 30% when ψ = 45° at the lumen distance *d*_lumen_ = 150 mm. In contrast, when changing the orientation of the deployed robot with the PM parallel to the actuated robot, *IoR* decreases remarkably with *F*_mean_ constant, as illustrated in fig. S11.

### Investigation of resistance in physiologically relevant conditions

**Fig. 3.**
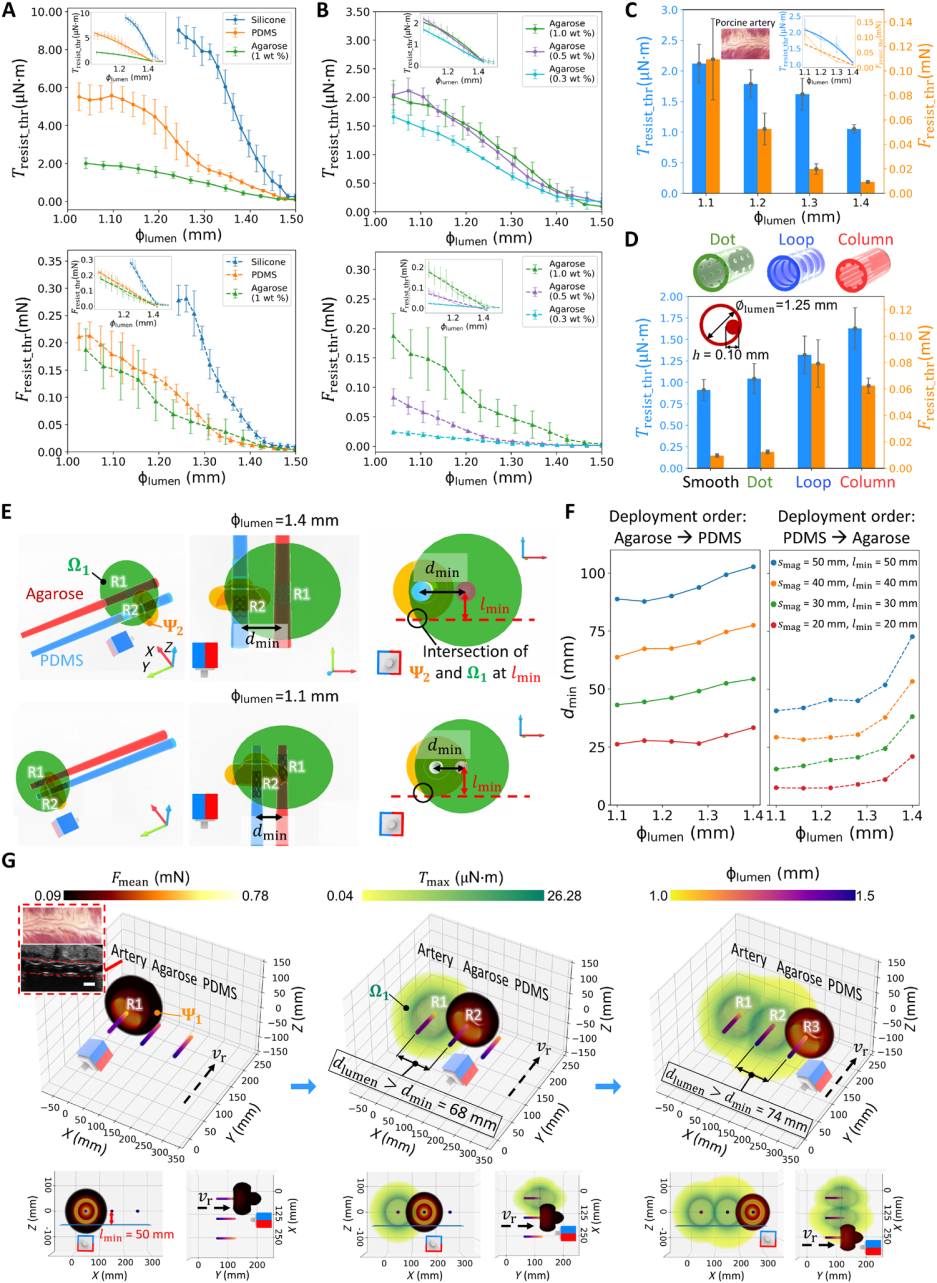
Investigation of the resistance variations in physiologically relevant lumen conditions. (**A** to **D**) Effects of the diameter (ϕ_lumen_), material type, stiffness, and surface pattern of lumens on *T*_resist_thr_ and *F*_resist_thr_. The ex vivo test results on porcine arteries are depicted in (C). A quadratic function and a linear equation are used to estimate the correlation between *T*_resist_thr_ and ϕ_lumen_, *F*_resist_thr_, and ϕ_lumen_, respectively, as presented in the subfigures of (A) to (C). (**E**) Investigation of the minimum lumen distance *d*_min_ allowing multirobot deployment based on the estimated resistance. When deploying two robots sequentially in lumens with different materials (1 wt % agarose and PDMS), *d*_min_ is determined where the boundaries of Ψ of robot R2 and Ω of robot R1 intersect at the minimum PM-lumen distance *l*_min_ along the *z* axis. (**F**) Theoretical determination of *d*_min_. Given two straight lumens with identical ϕ_lumen_, *d*_min_ is calculated based on the resistance estimation shown in (A) to (C), which can vary with the PM size (*s*_mag_), ϕ_lumen_, lumen material, and deployment order. (**G**) Experimental validation of the estimated *d*_min_. Three lumens of different materials (ex vivo porcine artery, 1 wt % agarose, and PDMS) are arranged in parallel at a distance surpassing *d*_min_. Three robots are sequentially deployed to the assigned lumen, and the corresponding Ω and Ψ concerning *s*_mag_=50 mm at the destination are illustrated. The robot inside the porcine artery is imaged by ultrasound imaging (scale bar, 1 mm). In all figures, error bars denote the standard deviation of measurements from one sample using four robots.

We use the online resistance estimation method to quantitatively investigate variations in *T*_resist_thr_ and *F*_resist_thr_ across diverse boundary conditions, including lumen material, stiffness, surface pattern, diameter, roundness, and fluid flow. In Fig. 3A, the relationship between *T*_resist_thr_, *F*_resist_thr_, and ϕ_lumen_ is studied within tapering lumens made from different synthetic materials, including silicone rubber, polydimethylsiloxane (PDMS), and agarose gel. As ϕ_lumen_ decreases, an augmented robot deformation induces an escalation in radial force *F*_n_, consequently amplifying friction *F*_friction_, leading to an increase in both *T*_resist_thr_ and *F*_resist_thr_. The quadratic and linear functions are used to correlate *T*_resist_thr_, *F*_resist_thr_, and ϕ_lumen_ as *F*_n_ exhibits linearity to ϕ_lumen_ (subfigures in Fig. 3, A to C), as detailed in the “Force modeling and analysis” section in Materials and Methods. Likewise, we investigate the influence of material stiffness on *T*_resist_thr_ and *F*_resist_thr_ with agarose gels of various stiffnesses. It is observed that the reduced stiffness corresponds to diminished *T*_resist_thr_ and *F*_resist_thr_ at the same initial ϕ_lumen_ (Fig. 3B), partially attributable to lumen expansion under the stress induced by robot deformation and a subsequently smaller *F*_n_ compared to stiffer lumens. Experimental assessments on ex vivo porcine arteries further validate the correlation between *T*_resist_thr_ and *F*_resist_thr_ concerning ϕ_lumen_ (Fig. 3C), where the robot is visualized by ultrasound imaging with the robotic system in fig. S12. Moreover, lumens exhibiting rough surfaces lead to an elevated friction coefficient, and consequently resistance augmentation (Fig. 3D). Lumen roundness also influences *T*_resist_thr_ and *F*_resist_thr_ (fig. S13), with their values falling between those for circular lumens with diameters equal to the long and short axes of the elliptical lumen. Furthermore, we have evaluated the effect of fluid viscosity and flow speed on resistance properties in fig. S14. For each lumen diameter, the increasing fluid viscosity and flow speed raise fluid drag, without substantially affecting *T*_resist_thr_ (comparing the light orange bars with others in fig. S14A) but substantially increasing *F*_resist_thr_ (comparing the light orange bars with others in fig. S14B).

Using the estimated correlation between *T*_resist_thr_, *F*_resist_thr_, and ϕ_lumen_, we investigate the minimum robot distance *d*_min_ for multirobot deployment, which depends on magnitudes of *T*_resist_thr_ and *F*_resist_thr_, *s*_mag_, geometry constraint, and robot deployment order. The investigation into *d*_min_ between two parallel straight lumens of different materials is presented in Fig. 3E. For deploying the target robot (R2), the placement of PM needs to satisfy two conditions: It should lie outside the Ω of the deployed robot (R1) and remain clear of the geometry constraint to prevent collisions with the sample, which necessitates the feasible Ψ = (Ψ_**2**_ − Ω_**1**_ − constraint), as illustrated in fig. S15. *d*_min_ is determined when the boundaries of Ψ_**2**_ and Ω_**1**_ intersect at the minimum PM-lumen distance *l*_min_. As the robot is actuated into a location with a reduced ϕ_lumen_, the ensuing rise in *T*_resist_thr_ leads to a smaller Ω, consequently reducing *d*_min_. In Fig. 3F, *d*_min_ is computed for two parallel straight lumens (PDMS and 1 wt % agarose gel) sharing the same ϕ_lumen_. Here, the deployment order plays an important role. If R1 is deployed into the PDMS lumen with larger resistance, its small Ω permits R2 to be in closer proximity to it, compared with the large Ω of the smoother agarose lumen. Moreover, a smaller *l*_min_ allows a small PM to actuate the robot with the same *F*_mag,*y*r_ as the large PM, and the reduced *s*_mag_ results in diminished Ωs of deployed robots and consequently decrease in *d*_min_. To validate the computed *d*_min_, three straight lumens, including the ex vivo porcine artery, agarose lumen, and PDMS lumen, are arranged parallel at intervals exceeding *d*_min_, where three robots are successfully deployed, as depicted in Fig. 3G and fig. S16.

### Deployment of multiple magnetic soft robots in 3D tubular structures

**Fig. 4.**
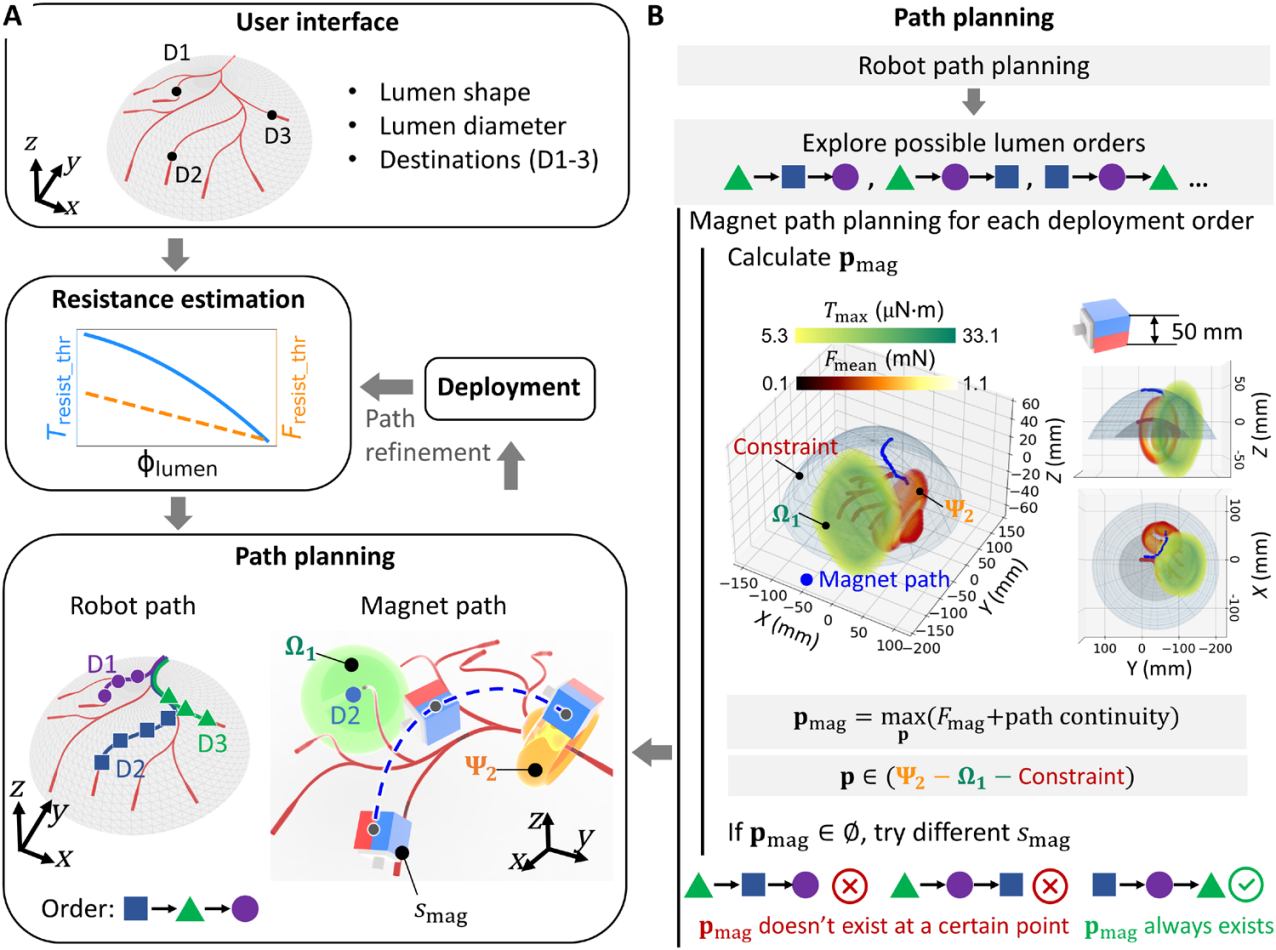
Schematic of the multirobot control process. (**A**) Overview of the multirobot control process. Initiating with lumen specifications, including shape, diameter, and robot destinations (D1 to D3) from the user interface, the lumen resistance estimation informs the path planning algorithm, which generates the robot paths, deployment order, and magnet paths and *s*_mag_ for each robot. In the path refinement process, the first robot is deployed based on the planning result [refer to the procedures in (B)], then *T*_resist_thr_ is re-estimated at its destination, refining magnet paths via the path planning algorithm for subsequent robots. This iterative process continues until the deployment of the last robot. (**B**) Schematic of the path planning algorithm. The deployment order is determined by iteratively exploring possible lumen orders until a feasible order is identified, ensuring the PM constantly remains outside the constraint and the Ω of the deployed robots while rotating at a position with the maximum *F*_mag,*y*r_ inside the Ψ of the target robot.

We present the overall deployment process of multiple soft robots within 3D lumen networks in Fig. 4A. Commencing with user-specified inputs through the interface, including the 3D lumen map, lumen diameters, and designated robot destinations, the pivot step is the resistance estimation, which can be performed offline or online. The offline estimation leverages a pre-acquired repository of resistance data of the target lumen material as the preknowledge. Conversely, in online estimation, the first robot is deployed to explore the resistance properties of the unknown environment and the resistance estimation is performed along the lumen. Using the resistance data, the path planning generates the robot paths, deployment order, and PM paths and *s*_mag_ for each robot, where the time for planning a single path varies between 1 and 6 s. To address the potential variation of lumen properties during navigation, the first robot is deployed based on the planning results, and *T*_resist_thr_ and *F*_resist_thr_ are estimated at its destination over a duration ranging from 20 to 90 s. The re-estimated resistance is then fed back to the planning module to refine magnet paths for subsequent robots. To enhance robustness against potential disturbances, magnitudes of *T*_resist_thr_ and *F*_resist_thr_ are scaled down and up, respectively, by coefficients such as 0.9 and 1.1. This iterative process continues as each robot is successively deployed, estimating *T*_resist_thr_ and *F*_resist_thr_ at their respective destinations, and feeding this information back into the planning algorithm, until the deployment of the final robot. Robots are initially placed at the entrance through a single catheterization, where they are magnetically or fluidically delivered within the catheter in sequence before following planned paths.

The path planning algorithm dictates the deployment order and trajectories of a specific-sized PM, ensuring its constant positioning outside the constraint and Ω of the deployed robots while rotating at a position maximizing *F*_mag,*y*r_ inside the feasible Ψ of the target robot. As demonstrated in Fig. 4B and fig. S17, the robot paths are first generated with the A* algorithm (*63*), yielding a list of discrete points at 2.5-mm intervals based on the 3D lumen map, after which the PM planning module explores the possible lumen deployment orders. For a given robot deployment order, the feasible Ψ is calculated along the robot path (fig. S15), and the PM position **p**_mag_ within it is computed to optimize both *F*_mean_ and path continuity. In cases where **p**_mag_ cannot be identified at a robot location with the specified *s*_mag_, the current deployment order is deemed unfeasible, and then another order is explored until a feasible order is found, ensuring the presence of **p**_mag_ along the entire robot path. Notably, changes in robot-lumen contact in branch areas affect the actuation strategy of a single robot passing the branch but do not influence the planning strategy for multirobot deployment. For single robot actuation, the lumen reaction force is leveraged to facilitate the robot to traverse branches by adjusting the PM rotational direction (*10*). For multirobot deployment, the planning strategy remains the same to compute the PM position to maximize the magnetic force for the actuated robot without avoid affecting deployed robots. This is because the Ωs of deployed robots same keep constant, while the Ψs of actuated robot expand given partial robot-lumen contact, making it easier for robot actuation using the same PM position as the fully contact case.

**Fig. 5.**
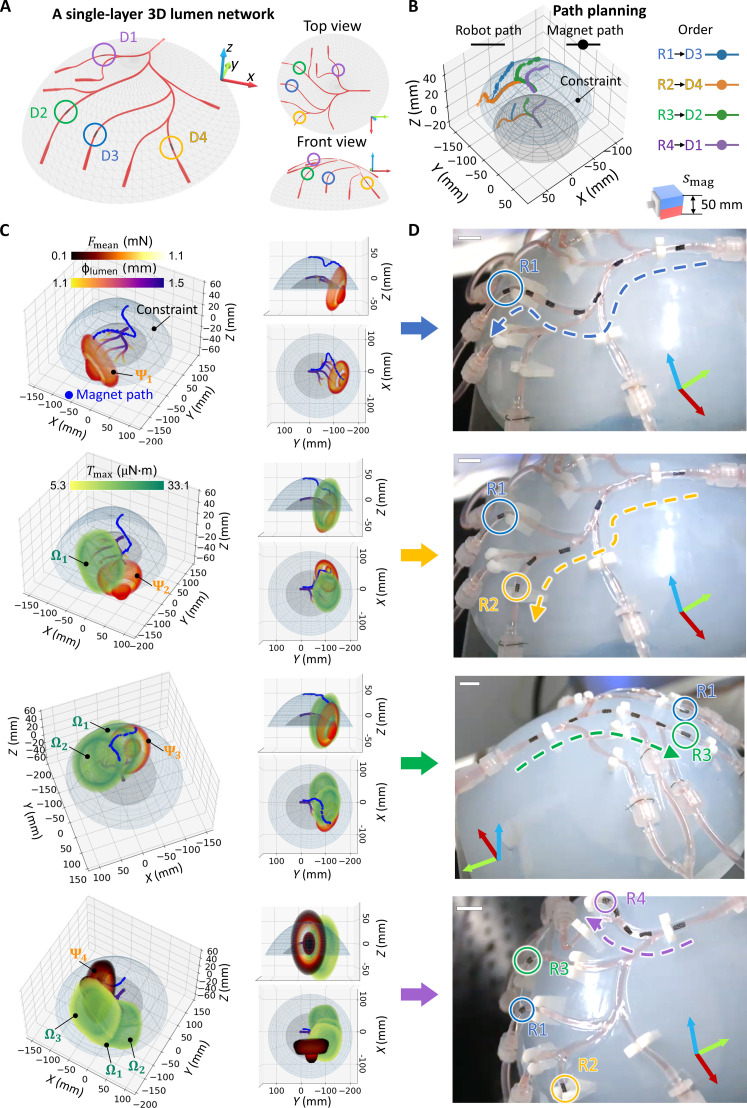
Deployment of four robots within a single-layer 3D lumen network. (**A**) Geometry of a single-layer 3D lumen network with targeted deployment destinations D1-D4. This phantom emulates the M4 distal vascular regions of the brain. (**B**) Path planning result for the multirobot deployment. (**C**) PM paths for the multirobot deployment. The magnet path, Ωs of deployed robots, and Ψ of the actuated robot at the destination are illustrated, where the PM constantly remains outside the Ω of the deployed robots while rotating at a position with the maximum *F*_mag,*y*r_ inside the Ψ of the actuated robot. (**D**) Sequential deployment of robots R1-R4 executing the planned paths. In all figures, scale bars are 10 mm.

**Fig. 6.**
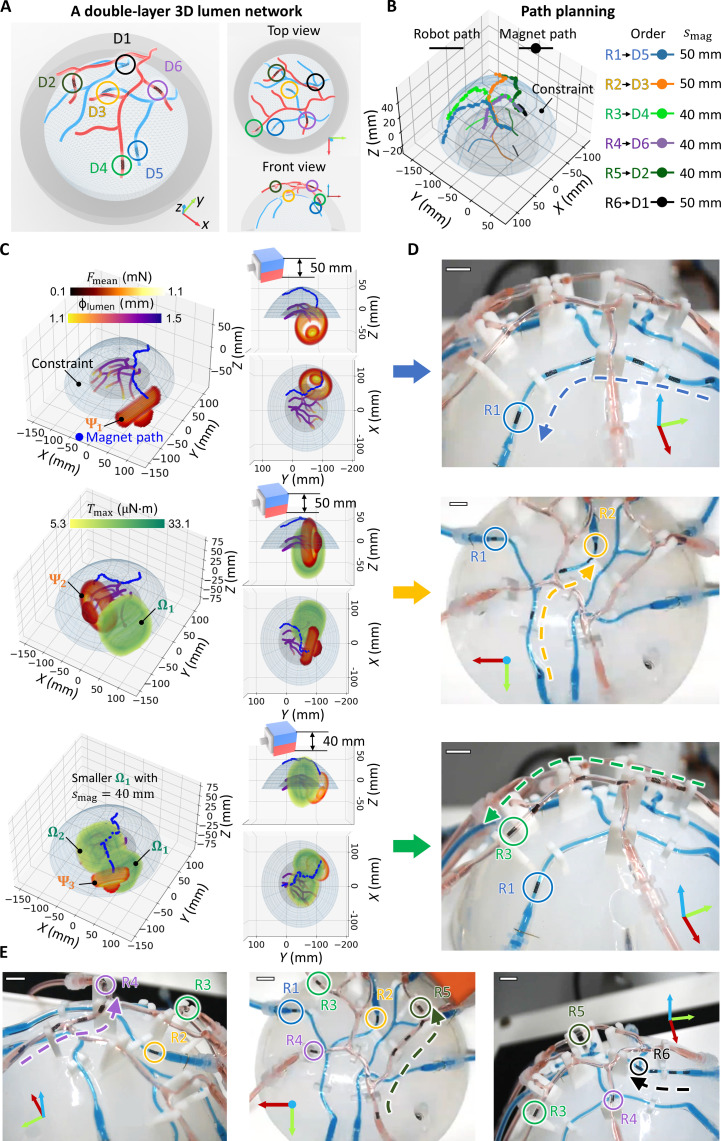
Deployment of six robots within a double-layer 3D lumen network. (**A**) Geometry of the double-layer 3D lumen network with targeted deployment destinations D1-D6 on two layers. (**B**) Path planning result for the multirobot deployment. (**C**) Magnet paths for the deployment of robot R1-3. The magnet path, Ωs of deployed robots, and Ψ of the actuated robot at the destination are illustrated. A 40-mm cubic PM is used to actuate R3 on the upper layer, minimizing the influence on R1 and R2 on the lower layer, while maximizing *F*_mag,*y*r_ on R3. The Ω of R1 under the 40-mm cubic PM is smaller than that under the 50-mm cubic PM. (**D**) Sequential deployment of robots R1-R3. (**E**) Sequential deployment of robots R4-R6. In all figures, scale bars are 10 mm.

The proposed multirobot deployment framework is experimentally validated using vessel phantoms simulating the M4 region of the human brain. In Fig. 5 and movie S1, four robots are deployed and then retrieved within a single-layer 3D lumen network. The paths of a 50-mm cubic PM, generated based on the estimated resistance of silicone rubber (Fig. 3A), facilitate supervised autonomous navigation for robots R1-4. The PM moves to the next position and orientation under manual command as the robot advances half its body length, monitored from two optical camera views. Expanding the deployment scope, Fig. 6 and movie S2 demonstrate the deployment of six robots within a multilayer 3D lumen network. The 50-mm cubic PM is used to actuate robots on the lower layer, leveraging its enhanced penetration capability given the geometry constraints that limit the minimum PM-robot distance. Furthermore, a 40-mm cubic PM is adopted to actuate upper-layer robots, minimizing the influence on lower-layer robots (reduced Ωs with 40-mm cubic PM compared with 50-mm cubic PM) and maximizing *F*_mag,*y*r_ on upper-layer robots. Here, independent robot control is achieved in 3D lumens despite the fluid flow disturbances, as visualized in movies S1 and S2. It should be noted that the maximum number of deployable robots depends on various factors such as network structure, lumen properties, targeted locations, and geometry constraints. In the multilayer 3D lumen network shown in Fig. 6, we have demonstrated the deployment of six robots, the majority of the maximum robot capacity of eight (fig. S18), validating the planning results.

### Operation in multiple sites under medical imaging

**Fig. 7.**
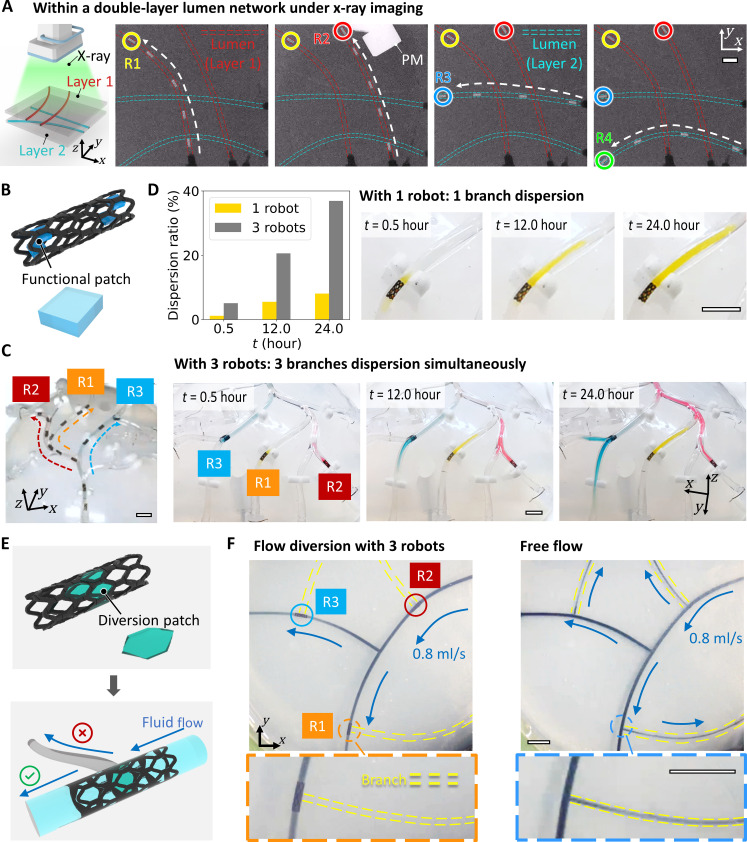
Multiple robot-based cargo delivery and flow diversion under x-ray medical imaging. (**A**) Deployment of four robots under x-ray cabinet imaging into a double-layer agarose gel phantom using a 20-mm cubic PM. (**B**) Integration of functional patches into the robot, exemplified with PDMS patches containing embedded pigments. (**C**) Deployment of three robots, each equipped with functional patches, within a 3D lumen network. (**D**) Enhanced cargo delivery efficacy with multiple robots. Concurrent multisite cargo delivery is achieved by the deployment of three robots at different locations, visually discernible by color variation, which is impossible for a single robot. Moreover, multiple robots enable the delivery of a larger cargo dosage, quantified by the dispersion ratio, denoting the percentage of lumen volume filled by the medicine relative to the entire tubular structure. (**E**) Robot design for flow diversion. Certain stent cells are sealed with PDMS as diversion patches, obstructing fluid flow into the lumen branch. (**F**) Multilocation flow diversion with three robots. The fluid flow into the branch lumens is blocked by three robots at diverse locations, where the branch lumen is denoted with the yellow dotted line. In all figures, scale bars are 10 mm.

To advance the proposed framework toward functional medical robotic systems, two key objectives need to be attained: realizing the medical functionalities of the robot in multiple sites and seamlessly integrating it with the medical imaging system. First, the multirobot deployment is conducted under the real-time x-ray cabinet imaging (frame rate, 10 Hz) with a 5-DOF robotic system (fig. S19), where four robots are sequentially deployed into a double-layer lumen network with a 20-mm PM, as shown in Fig. 7A and movie S3. The distinct contrast between the robot and background materials underscores its potential for concurrent multilocation therapeutics in vivo.

The incorporation of functional modules transforms the robot into a wireless medical device, showcasing two proof-of-concept functions, multisite cargo delivery, and multisite flow diversion. For targeted cargo delivery, tiny functional patches are affixed to the single beam (Fig. 7B), exemplified with PDMS patches containing embedded pigments. As shown in Fig. 7C and movie S4, three robots with functional patches are sequentially actuated to the target locations and gradually release various pigments into the surrounding area over 24 hours. The advantages of cargo delivery with multiple robots are manifold: simultaneous cargo release at multiple locations, a substantial increase in total delivered cargo amount, and the ability to use various cargo types. Escalated cargo delivery efficacy is observed in Fig. 7D and movie S4, evaluated by the percentage of lumen volume filled by the dye relative to the entire tubular structure (dispersion ratio), with various pigments dispersing to a larger area.

Multiple robots can serve as actively controlled flow diverters to regulate fluid flow distributively. For instance, blocking the blood flow of multiple vessels into tumors to impede their growth could be achieved, which is unattainable with a single robot. The proof-of-concept robot-based flow diverter is achieved by sealing certain stent cells with PDMS to obstruct the flow, as illustrated in Fig. 7E. Three robots are deployed to their destinations and prevent the flow from entering side branches in Fig. 7F and movie S5. Notably, diverted flow volume could be tuned by porosity adjustment with the robot rotation, actively modifying the ratio between the uncovered area by the sealed cells and the total area. Future efforts could focus on optimizing flow diverter designs and conducting further investigations into diversion efficacy across various physiological environments.

## DISCUSSION

Deploying multiple magnetic miniature soft robotic devices under physiologically relevant conditions presents challenges arising from the influence of the external field on all agents and interactions with the surrounding environment. To address this issue, we have reported a framework leveraging the interplay between magnetic actuation, shape-adaptive robotic design, and heterogeneity in interactive resistance, enabling multiple robot deployment in 3D lumen networks with pulsatile fluid flow. An online resistance estimation method has been used to quantitatively investigate resistance changes under various boundary conditions, including lumen material, stiffness, surface pattern, diameter, roundness, and fluid flow. Furthermore, two quantified critical regions, the influence and actuation regions, that affect the locomotion behaviors have been proposed and computed, with which a path planning method has been developed to generate the trajectory of a PM for robot deployment in a complex 3D lumen network. Validation of the framework includes deploying multiple robots into ex vivo tissue and synthetic lumens, into a single-layer 3D lumen network, and into a multilayer 3D tubular structure. Last, multilocation cargo delivery, flow diversion, and deployment of multiple robots under x-ray cabinet imaging have been conducted to demonstrate the potential enhancement of biomedical functions in both spatial and temporal domains.

Compared with existing decoupled control methods for magnetic robots focusing on 2D environments without flow, our system promises a much-enhanced number of magnetic soft robots (>5) deployed within 3D lumens with fluidic flow at the millimeter scale, boosting the efficiency of therapeutic interventions and opening the door for spatially distributed medical functions. The proposed method supports treatment of multisite diseases (typically >3) in distal cortical artery segments, such as brain tumors (*28*, *29*), aneurysms (*33*, *34*), and strokes (*40*–*42*), where the targeted sites spaced over 15 mm apart inside the human brain (average size, 167 mm × 140 mm × 93 mm) can be potentially accessed. For instance, six robots can be deployed to multiple blood vessels connected to a brain tumor, enabling coordinated drug delivery and embolization to improve the treatment efficacy. Further details on the benefits of using multiple robots are summarized in table S1. It is worth noting that the maximum magnitude of the magnetic field in this work is less than 200 mT, which is safe for patients (*1*).

The proposed multirobot control strategy can be expanded by integrating additional functions and adapting it to various applications. Beside the demonstrated concurrent execution of targeted cargo delivery and flow diversion, the incorporation of metallic materials could enable distributed on-demand localized heating (*20*). In addition, the integration of onboard electronic modules could facilitate the distributed sensing of physiological properties, such as temperature (*37*), flow speed (*36*), and electrophysiological parameters (*38*). Notably, using the online resistance estimation method, the robot body can potentially function as a sensor to monitor the resistance property of surrounding tissues for understanding tissue biomechanics in vivo during disease development and providing feedback information for therapeutic solutions (*29*). Moreover, the proposed method could extend its applicability to other magnetically controlled soft robots (*21*, *27*) and commercial medical devices (*64*, *65*). For instance, commercial robotic capsules have been widely used for endoscopy (*64*) and drug delivery (*65*) in the gastrointestinal tract. The capsule fully contacts the tissue surface and requires sufficient actuation to overcome the mucus barrier for locomotion, based on which multiple capsules could be deployed with the proposed framework. In addition, the spatially distributed flow diversion capability could be harnessed to control the fluidic flow in microfluid devices (*66*, *67*).

The multirobot deployment capability can be further augmented from the following aspects. First, we have quantitatively investigated the influence of lumen properties on the resistive torque threshold (*T*_resist_thr_) and force threshold (*F*_resist_thr_), and the effect of magnet size and orientation on magnetic torque and force. These resistive thresholds and magnetic actuation dictate the size and shape of the influence region (Ω) and actuation region (Ψ), which further influences the number of deployable robots within a region. To increase the number of robots, the incorporation of active locking structures to the robot can be adopted, such as the stent structure constructed from shape memory polymer (*68*) or jig-assisted surface-anchoring structure (*26*). With the on-demand-triggered locking module, a larger robot-tissue resistance can be achieved, and a reduced Ω and an enlarged feasible Ψ can be acquired, which enables the deployment of more robots and enhances robustness to substantial resistance variations in unstructured biological environments. It is important to acknowledge that the resistance arising from soft-bodied interactions is a complicated topic associated with various factors, such as surface structure, material properties, fluid dynamics, and applied load force. A more intricate model detailing the soft surface contact between the robot and the lumen can offer insights into the robot dynamics, facilitating a more efficient model-based control strategy.

The use of multiple PMs can further improve deployment efficiency. As illustrated in fig. S20, coordinated PMs enables the exertion of a larger magnetic torque and force to actuate the desired robot to overcome tissue barriers, while magnetic field neutralization eliminates interference with nontargeted robots. Using this strategy, multi-PM coordination increases robot density by tactically adjusting their positions, orientations, rotation directions, sizes, and phase differences (fig. S21, A to C). Furthermore, multiple robots can be actuated simultaneously for enhanced deployment speed (fig. S21, D to F), compared with the sequential deployment with a single PM. However, this approach presents challenges, such as increased control complexity of optimizing numerous variables, high localization precision requirements, and less intuitive control without Ω and Ψ. In addition, we have studied the multirobot deployment within 3D lumens on an ellipsoidal surface resembling the M4 region of the human brain, and deploying multiple robots within lumens on increasingly irregular surfaces can be further investigated. Last but not least, more quantitative analyses of the resistance properties (friction and fluid drag) for specific arteries, the 3D mapping of blood vessel networks, and robot localization under medical imaging are imperative for an autonomous medical robotic system towards in vivo medical applications.

## MATERIALS AND METHODS

### Magnetic actuation system for multirobot control

The 6-DOF robotic system for multirobot control in 3D lumens includes a cubic PM (N45, IMPLOTEX GmbH), a NEMA 17 stepper motor for magnet rotation, and a 7-DOF robotic arm (Panda, Franka Emika GmbH). The cubic PMs are 20, 30, 40, and 50 mm in size, with maximum magnetic field strengths of 103, 141, 154, and 187 mT at minimum allowable robot-magnet distances of 20, 25, 35, and 41 mm, respectively. Communication software was developed using the Robot Operating System (ROS Noetic), organized into four primary nodes: a motion command generator node, a step motor controller node, an arm controller node, and a path planning node. During operation, motion commands are sourced from both manual inputs and via-points along the magnet path. Upon manual commands issued by the joystick, the rotating PM automatically moves to the subsequent position and orientation generated by the path planning node. Manual commands are transferred to the arm controller node and the step motor controller. The integrated system facilitates 6-DOF spatial manipulation around the end-effector. Further, another robotic arm (Panda, Franka Emika GmbH) with an ultrasound probe (Vevo 3100, FUJIFILM Visualsonics Inc.) was used for robot deployment inside the porcine artery.

For multiple robot deployment under x-ray cabinet imaging (XPERT 80, KUBTEC Scientific), a 20-mm cubic magnet (N45, IMPLOTEX GmbH) was controlled by two translational motorized stages (LTS300/M, Thorlabs Inc.) with a stepper motor (535-0372, RS Components GmbH) and a servo motor (SKU 900-00360, Parallax Inc.) mounting on them. The height of the sample was adjusted with a translational motorized stage (LTS300/M, Thorlabs Inc.). These components formed a 5-DOF robotic system.

### Force modeling and analysis

The robot overcomes the resistance to move forward under the magnetic actuation, including the magnetic torque around the *y*_r_ axis, *T*_mag,*y*r_, and magnetic force along the *y*_r_ axis, *F*_mag,*y*r_. *T*_mag,*y*r_ and *F*_mag,*y*r_ are computed with the dipole model (*24*, *62*) as

$$T_{\text{mag},yr}=\left[m_{r}\times B\left(p_{a}^{r}\right)\right]∙{\hat{\omega }}_{r}$$ (1)

$$F_{\text{mag},yr}=\left[\left(m_{r}∙\nabla \right)B\left(p_{a}^{r}\right)\right]∙{\hat{\omega }}_{r}$$ (2)

where **m**_r_ is the magnetic moment of the robot, $B\left(p_{a}^{r}\right)$ is the magnetic flux density generated by the PM with the magnetic moment of **m**_a_, ${\hat{\omega }}_{r}$ is the unit vector along the *y*_r_ axis and the rotation axis of the robot, and $p_{a}^{r}$ is the vector from the PM to the robot. $B\left(p_{a}^{r}\right)$ is computed with

$$B\left(p_{a}^{r}\right)=\frac{\mu_{0}Hm_{a}}{4\pi {\left\|p_{a}^{r}\right\|}^{3}}$$ (3)

where *H* = $3{\hat{p}}_{a}^{r}{\left({\hat{p}}_{a}^{r}\right)}^{T}-I$, μ_0_ = 4π × 10^−7^ N · A^−2^ is the permeability of free space, **m**_a_ is the magnetic moment of the PM, and *I* ∈ ℝ^3×3^ is the identity matrix. The magnetic field magnitude generated by the PM fluctuates in an elliptical fashion with the rotation axis ${\hat{\omega }}_{b}=\hat{H^{-1}{\hat{\omega }}_{a}}$, and ${\hat{\omega }}_{a}$ is the rotation axis of the PM (fig. S2). The projection of the ellipsis magnetic field onto the *x*_r_−*z*_r_ plane is also an ellipsis, and the robot rotation can be modeled as

$$T_{\text{mag},yr}-T_{\text{resist}}=J_{yr}\frac{d^{2}\varphi_{r}}{dt^{2}}$$ (4)

where *T*_resist_ is the resistive force around the *y*_r_ axis, *J*_*y*r_ is the moment of inertia around the *y*_r_ axis, and φ_r_ is the rotation angle of the robot around the *y*_r_ axis. Since *J*_*y*r_ is around 1.5 × 10^−12^ kg · m^2^ and *T*_resist_ is larger than 1 × 10^−6^ N · m, *T*_mag,*y*r_ is approximately equal to *T*_resist_, based on which the angle difference ∆φ_r_ between **m**_r_ and **B***_xz_*, and *F*_mag,*y*r_ are further computed (fig. S2). The robot translation is modeled as

$$F_{\text{mag},yr}-F_{\text{resist}}=m_{r}\frac{d^{2}l_{r}}{dt^{2}}$$ (5)

where *F*_resist_ is the resistive force along the *y*_r_ axis, *m*_r_ is the robot mass, and *l*_r_ is the robot displacement along the *y*_r_ axis.

As illustrated in fig. S22, resistance is modeled with friction and fluid drag as

$$T_{\text{resist}}=\left(F_{\text{friction}}^{r}+F_{\text{fluid}}^{r}\right)R_{d}$$ (6)

$$F_{\text{resist}}=F_{\text{friction}}^{t}+{\mathrm{bF}}_{\text{fluid}}^{t}$$ (7)

where $F_{\text{fluid}}^{r}$, $F_{\text{fluid}}^{t}$, and *R*_d_ are fluid drag on the *x*_r_−*z*_r_ plane, fluid drag along the *y*_r_ axis, robot radius, respectively, *b* is a parameter equal to 1 or −1 when the robot moves against or along the flow, and

$$F_{\text{friction}}^{r}=\left[\mu_{\|}\text{cos}\left(\alpha \right)+\mu_{⊥}\text{sin}\left(\alpha \right)\right]\left(F_{n}-F_{\text{adhesion}}\right)$$ (8)

$$F_{\text{friction}}^{t}=\left[\mu_{\|}\text{sin}\left(\alpha \right)-\mu_{⊥}\text{cos}\left(\alpha \right)\right]\left(F_{n}-F_{\text{adhesion}}\right)$$ (9)

In Eqs. 8 and 9, *F*_n_ is the radial force, *F*_adhesion_ is the adhesion, α is the helix angle (8°), and μ_⊥_ and μ_‖_ are the coefficient of friction perpendicular to and parallel to the helix, respectively. With the force analysis on the simplified stent geometry (*10*), *F*_n_ can be expressed as

$$F_{n}=10\frac{3E_{r}l_{b}∆l_{c}}{{\left(l_{b}\right)}^{3}}\text{tan}\left(\frac{\pi }{3}\right)$$ (10)

where *E*_r_ is the Young’s modulus of the robot material, *I*_b_ is the second moment of area for the cross section of a single beam on the composing diamond-shaped cell, *l*_b_ is the length of the single beam, and ∆*l*_c_ is the change in distance of the two facing beams of the cell under deformation, which is linear to *R*_d_. Assuming that the friction coefficients, adhesion, and fluid drag are constant, quadratic and linear functions can be used to correlate *T*_resist_, *F*_resist_, and *R*_d_ as *F*_n_ exhibits linearity to *R*_d_. The fluid drag is modeled as *F*_fluid_ = 0.5ρ*u*^2^*c*_d_*A*, where ρ is the mass density of the fluid, *u* is the flow velocity relative to the robot, *A* is the reference area, and *c*_d_ is the drag coefficient relating to the robot geometry and Reynolds number (*69*). Increasing fluid viscosity leads to a larger *c*_d_ and consequently enhanced *F*_fluid_, while higher *u* also increases *F*_fluid_. Since *T*_resist_ and *F*_resist_ vary given a static robot, the resistive torque threshold *T*_resist_thr_ and the resistive force threshold *F*_resist_thr_ are approximated as *T*_resist_ and *F*_resist_ during the robot locomotion.

### Preparation of synthetic materials and phantoms

The agarose gel samples with lumens inside are illustrated in fig. S23. The positive mold with the desired lumen shape and diameter was printed with the 3D printer (Form 3, Formlabs Inc.). Agarose powder (BioReagents A9539, Sigma-Aldrich Co.) was dissolved in deionized water at 90°C with continuous stirring until complete dissolution. The solution was then boiled for 5 min and poured into the petri dish where the positive mold was inside. After cooling to room temperature (approximately 24°C) for 30 min, the positive mold was extracted. Similarly, PDMS elastomer with a weight ratio of 8:1 between the monomer to the cross-linker was poured to make the PDMS samples with lumens inside. The customized 3D lumen phantoms were produced by the Trandomed Co. Ltd., using silicone rubber as the phantom material. The blood analog for all the quantitative analyses in phantoms was the glycerol/deionized water mixture, and the volume ratio was 44 to 65.

### Preparation of animal organs for the ex vivo test

The porcine hearts were obtained as animal by-products (registration number: DE 08 111 1008 21), with permits and registration issued by the authorities for official food control, consumer protection, and veterinary services of the state capital in Stuttgart. Following the permit guidelines, a register of biomaterial usage is maintained, and postexperiment, the animal by-products are pressure sterilized. Coronary arteries for locomotion experiments and resistance tests were extracted from fresh porcine hearts within 48 hours of slaughter and stored at 4°C (Slaughterhouse Ulm, Germany, and Gourmet Compagnie GmbH, Germany). Tissues were cleaned with phosphate-buffered saline (PBS) before testing. For all ex vivo demonstrations in organs, PBS (pH = 7.4, Gibco, Thermo Fisher Scientific) was pumped to the porcine arteries at a flow rate of 10 to 12 ml/min.

### Quantification of the resistance properties for the synthetic and ex vivo animal materials

Tapering lumens made of synthetic materials (PDMS, silicone rubbers, and agarose gel) were prepared to quantitatively investigate the resistance properties, with the lumen diameter ranging from 1.5 to 1.0 mm. The robots were actuated to multiple locations along the lumen with various diameters, and the lumen diameter at the robot center was used to correlate with the resistance. The resistance estimation procedure in Fig. 2A was repeatedly performed at these locations. Similarly, the robots were deployed to the porcine artery, and their behavior was monitored by the ultrasound imaging system. For the validation of the influence, rotation, and actuation regions, the resistance estimation was first conducted, and then these regions were computed. Subsequently, the PM relocated along the *y*_r_ axis and approached the robot along the *z*_r_ axis, during which the robot behavior was observed and compared with the computed region.

### Preparation of the proof-of-concept functional modules

The patches for the proof-of-concept cargo delivery were fabricated using the procedures in fig. S24. First, PDMS (mixture ratio, monomer: cross-linker = 10:1 by weight, Sylgard 184, Dow Inc.) and pigments were mixed at a 5:1 ratio by weight and then poured onto apoly(methyl methacrylate) substrate with 200-μm-thick spacers, against which a razor blade was scraped for the control of the sheet thickness. The scraped mixture was cured at 90°C on a hot plate for 90 min. The cured sheet was then cut into a 0.3 mm × 0.3 mm rectangular patch using a laser machine (LPKF ProtoLaser U3, LPKF Laser & Electronics AG). Subsequently, the patches were bonded with the robot body with PDMS of the same mixture ratio. The flow diverters were fabricated by filling the stent cell with PDMS (mixture ratio, monomer: cross-linker = 20:1 by weight, Sylgard 184, Dow Inc.), and then the filled PDMS was cured at 90°C on a hot plate for 90 min.

### Collection of visual data with optical and medical imaging

For quantitative experiments assessing robot locomotion and functionality, data were captured using two Blackfly S USB3 cameras (Teledyne FLIR LLC) and SpinView 2.4.0 software. Ultrasound-based inspection of the robot in porcine coronary artery ex vivo experiments used the B-mode of a Vevo 3100 medical ultrasound machine (FUJIFILM Visualsonics Inc). Imaging data were transferred to a PC via a DVI2USB 3.0 video grabber (Epiphan Systems Inc) and a ROS-compatible video streaming driver. X-ray inspection was conducted with a commercial x-ray cabinet imaging system (XPERT 80, KUBTEC Scientific) and KubtecNC 3.0.0.0 software.

## Supplementary Materials

### The PDF file includes:

Figs. S1 to S24 Table S1 Legends for movies S1 to S5 References

## References

[1] M. Sitti, *Mobile microrobotics* (MIT Press, Cambridge, MA, 2017).

[2] M. Cianchetti, C. Laschi, A. Menciassi, P. Dario, Biomedical applications of soft robotics. Nat. Rev. Mater. 3, 143–153 (2018).

[3] T. Wang, Y. Wu, E. Yildiz, S. Kanyas, M. Sitti, Clinical translation of wireless soft robotic medical devices. Nat. Rev. Bioeng. 2, 470–485 (2024).

[4] M. Z. Miskin, A. J. Cortese, K. Dorsey, E. P. Esposito, M. F. Reynolds, Q. Liu, M. Cao, D. A. Muller, P. L. McEuen, I. Cohen, Electronically integrated, mass-manufactured, microscopic robots. Nature 584, 557–561 (2020). 32848225 10.1038/s41586-020-2626-9

[5] S. Palagi, A. G. Mark, S. Y. Reigh, K. Melde, T. Qiu, H. Zeng, C. Parmeggiani, D. Martella, A. Sanchez-Castillo, N. Kapernaum, Structured light enables biomimetic swimming and versatile locomotion of photoresponsive soft microrobots. Nat. Mater. 15, 647–653 (2016). 26878315 10.1038/nmat4569

[6] M. D. Brown, B. T. Cox, B. E. Treeby, Stackable acoustic holograms. Appl. Phys. Lett. 116, 261901 (2020).

[7] Z. Ma, K. Melde, A. G. Athanassiadis, M. Schau, H. Richter, T. Qiu, P. Fischer, Spatial ultrasound modulation by digitally controlling microbubble arrays. Nat. Commun. 11, 4537 (2020). 32913270 10.1038/s41467-020-18347-2 PMC7484750

[8] B. Hao, X. Wang, Y. Dong, M. Sun, C. Xin, H. Yang, Y. Cao, J. Zhu, X. Liu, C. Zhang, Focused ultrasound enables selective actuation and Newton-level force output of untethered soft robots. Nat. Commun. 15, 5197 (2024). 38890294 10.1038/s41467-024-49148-6 PMC11189400

[9] W. Hu, G. Z. Lum, M. Mastrangeli, M. Sitti, Small-scale soft-bodied robot with multimodal locomotion. Nature 554, 81–85 (2018). 29364873 10.1038/nature25443

[10] T. Wang, H. Ugurlu, Y. Yan, M. Li, M. Li, A.-M. Wild, E. Yildiz, M. Schneider, D. Sheehan, W. Hu, Adaptive wireless millirobotic locomotion into distal vasculature. Nat. Commun. 13, 4465 (2022). 35915075 10.1038/s41467-022-32059-9 PMC9343456

[11] M. Sitti, Miniature soft robots—Road to the clinic. Nat. Rev. Mater. 3, 74–75 (2018).

[12] M. Sitti, H. Ceylan, W. Hu, J. Giltinan, M. Turan, S. Yim, E. Diller, Biomedical applications of untethered mobile milli/microrobots. Proc. IEEE Inst. Electr. Electron Eng. 103, 205–224 (2015). 27746484 10.1109/JPROC.2014.2385105 PMC5063027

[13] M. Li, A. Pal, A. Aghakhani, A. Pena-Francesch, M. Sitti, Soft actuators for real-world applications. Nat. Rev. Mater. 7, 235–249 (2022). 35474944 10.1038/s41578-021-00389-7 PMC7612659

[14] S. Lee, S. Kim, S. Kim, J. Y. Kim, C. Moon, B. J. Nelson, H. Choi, A capsule-type microrobot with pick-and-drop motion for targeted drug and cell delivery. Adv. Healthc. Mater. 7, e1700985 (2018). 29460365 10.1002/adhm.201700985

[15] J. Tang, C. Yao, Z. Gu, S. Jung, D. Luo, D. Yang, Super-soft and super-elastic DNA robot with magnetically driven navigational locomotion for cell delivery in confined space. Angew. Chem. Int. Ed. Engl. 59, 2490–2495 (2020). 31769147 10.1002/anie.201913549

[16] B. Wang, K. F. Chan, K. Yuan, Q. Wang, X. Xia, L. Yang, H. Ko, Y.-X. J. Wang, J. J. Y. Sung, P. W. Y. Chiu, Endoscopy-assisted magnetic navigation of biohybrid soft microrobots with rapid endoluminal delivery and imaging. Sci. Robot. 6, eabd2813 (2021). 34043547 10.1126/scirobotics.abd2813

[17] Y. Kim, E. Genevriere, P. Harker, J. Choe, M. Balicki, R. W. Regenhardt, J. E. Vranic, A. A. Dmytriw, A. B. Patel, X. Zhao, Telerobotic neurovascular interventions with magnetic manipulation. Sci. Robot. 7, eabg9907 (2022). 35417201 10.1126/scirobotics.abg9907 PMC9254892

[18] X. Liu, L. Wang, Y. Xiang, F. Liao, N. Li, J. Li, J. Wang, Q. Wu, C. Zhou, Y. Yang, Magnetic soft microfiberbots for robotic embolization. Sci. Robot. 9, eadh2479 (2024). 38381840 10.1126/scirobotics.adh2479

[19] J. Law, X. Wang, M. Luo, L. Xin, X. Du, W. Dou, T. Wang, G. Shan, Y. Wang, P. Song, Microrobotic swarms for selective embolization. Sci. Adv. 8, eabm5752 (2022). 35857830 10.1126/sciadv.abm5752 PMC9299543

[20] R. H. Soon, Z. Yin, M. A. Dogan, N. O. Dogan, M. E. Tiryaki, A. C. Karacakol, A. Aydin, P. Esmaeili-Dokht, M. Sitti, Pangolin-inspired untethered magnetic robot for on-demand biomedical heating applications. Nat. Commun. 14, 3320 (2023). 37339969 10.1038/s41467-023-38689-x PMC10282021

[21] C. Wang, Y. Wu, X. Dong, M. Armacki, M. Sitti, In situ sensing physiological properties of biological tissues using wireless miniature soft robots. Sci. Adv. 9, eadg3988 (2023). 37285426 10.1126/sciadv.adg3988 PMC7614673

[22] J. Han, X. Dong, Z. Yin, S. Zhang, M. Li, Z. Zheng, M. C. Ugurlu, W. Jiang, H. Liu, M. Sitti, Actuation-enhanced multifunctional sensing and information recognition by magnetic artificial cilia arrays. Proc. Natl. Acad. Sci. U.S.A. 120, e2308301120 (2023). 37792517 10.1073/pnas.2308301120 PMC10589697

[23] B. Xiao, Y. Xu, S. Edwards, L. Balakumar, X. Dong, Sensing mucus physiological property in situ by wireless millimeter-scale soft robots. Adv. Funct. Mater. 34, 2307751 (2024). 10.1002/adfm.202307751 PMC11845219 39990597

[24] J. J. Abbott, E. Diller, A. J. Petruska, Magnetic methods in robotics. Annu. Rev. Control Robot. Auton. Syst. 3, 57–90 (2020).

[25] Y. Wu, X. Dong, J.-k. Kim, C. Wang, M. Sitti, Wireless soft millirobots for climbing three-dimensional surfaces in confined spaces. Sci. Adv. 8, eabn3431 (2022). 35622917 10.1126/sciadv.abn3431 PMC9140972

[26] J. Zhang, Z. Ren, W. Hu, R. H. Soon, I. C. Yasa, Z. Liu, M. Sitti, Voxelated three-dimensional miniature magnetic soft machines via multimaterial heterogeneous assembly. Sci. Robot. 6, eabf0112 (2021). 34043568 10.1126/scirobotics.abf0112 PMC7612277

[27] Z. Ren, R. Zhang, R. H. Soon, Z. Liu, W. Hu, P. R. Onck, M. Sitti, Soft-bodied adaptive multimodal locomotion strategies in fluid-filled confined spaces. Sci. Adv. 7, eabh2022 (2021). 34193416 10.1126/sciadv.abh2022 PMC8245043

[28] J. R. McFaline-Figueroa, E. Q. Lee, Brain tumors. Am. J. Med. 131, 874–882 (2018). 29371158 10.1016/j.amjmed.2017.12.039

[29] T. Tunthanathip, K. Kanjanapradit, S. Ratanalert, N. Phuenpathom, T. Oearsakul, A. Kaewborisutsakul, Multiple, primary brain tumors with diverse origins and different localizations: Case series and review of the literature. J. Neurosci. Rural Pract. 9, 593–607 (2018). 30271057 10.4103/jnrp.jnrp_82_18 PMC6126305

[30] M. Nasor, W. Obaid, Detection and localization of early-stage multiple brain tumors using a hybrid technique of patch-based processing, k-means clustering and object counting. Int. J. Biomed. Imaging 2020, 9035096 (2020). 32494290 10.1155/2020/9035096 PMC7199552

[31] K. Ikeda, H. Wakimoto, T. Ichikawa, S. Jhung, F. H. Hochberg, D. N. Louis, E. A. Chiocca, Complement depletion facilitates the infection of multiple brain tumors by an intravascular, replication-conditional herpes simplex virus mutant. J. Virol. 74, 4765–4775 (2000). 10775615 10.1128/jvi.74.10.4765-4775.2000 PMC111999

[32] J. Rahmer, C. Stehning, B. Gleich, Spatially selective remote magnetic actuation of identical helical micromachines. Sci. Robot. 2, eaal2845 (2017). 33157862 10.1126/scirobotics.aal2845

[33] S. Juvela, Risk factors for multiple intracranial aneurysms. Stroke 31, 392–397 (2000). 10657411 10.1161/01.str.31.2.392

[34] K. Mizoi, J. Suzuki, T. Yoshimoto, Surgical treatment of multiple aneurysms: Review of experience with 372 cases. Acta Neurochir. 96, 8–14 (1989). 2929394 10.1007/BF01403489

[35] J. Hu, H. Albadawi, B. W. Chong, A. R. Deipolyi, R. A. Sheth, A. Khademhosseini, R. Oklu, Advances in biomaterials and technologies for vascular embolization. Adv. Mater. 31, e1901071 (2019). 31168915 10.1002/adma.201901071 PMC7014563

[36] D.-H. Kim, N. Lu, R. Ghaffari, Y.-S. Kim, S. P. Lee, L. Xu, J. Wu, R.-H. Kim, J. Song, Z. Liu, Materials for multifunctional balloon catheters with capabilities in cardiac electrophysiological mapping and ablation therapy. Nat. Mater. 10, 316–323 (2011). 21378969 10.1038/nmat2971 PMC3132573

[37] D.-H. Kim, R. Ghaffari, N. Lu, S. Wang, S. P. Lee, H. Keum, R. D’Angelo, L. Klinker, Y. Su, C. Lu, Electronic sensor and actuator webs for large-area complex geometry cardiac mapping and therapy. Proc. Natl. Acad. Sci. U.S.A. 109, 19910–19915 (2012). 23150574 10.1073/pnas.1205923109 PMC3523871

[38] M. Han, L. Chen, K. Aras, C. Liang, X. Chen, H. Zhao, K. Li, N. R. Faye, B. Sun, J.-H. Kim, Catheter-integrated soft multilayer electronic arrays for multiplexed sensing and actuation during cardiac surgery. Nat. Biomed. Eng. 4, 997–1009 (2020). 32895515 10.1038/s41551-020-00604-w PMC8021456

[39] B. H. Kim, K. Li, J.-T. Kim, Y. Park, H. Jang, X. Wang, Z. Xie, S. M. Won, H.-J. Yoon, G. Lee, Three-dimensional electronic microfliers inspired by wind-dispersed seeds. Nature 597, 503–510 (2021). 34552257 10.1038/s41586-021-03847-y

[40] J. L. Saver, R. Chapot, R. Agid, A. E. Hassan, A. P. Jadhav, D. S. Liebeskind, K. Lobotesis, D. Meila, L. Meyer, G. Raphaeli, Thrombectomy for distal, medium vessel occlusions: A consensus statement on present knowledge and promising directions. Stroke 51, 2872–2884 (2020). 32757757 10.1161/STROKEAHA.120.028956

[41] A. R. Xavier, A. M. Siddiqui, J. F. Kirmani, R. A. Hanel, A. M. Yahia, A. I. Qureshi, Clinical potential of intra-arterial thrombolytic therapy in patients with acute ischaemic stroke. CNS Drugs 17, 213–224 (2003). 12665395 10.2165/00023210-200317040-00001

[42] J. A. Grossberg, L. C. Rebello, D. C. Haussen, M. Bouslama, M. Bowen, C. M. Barreira, S. R. Belagaje, M. R. Frankel, R. G. Nogueira, Beyond large vessel occlusion strokes: Distal occlusion thrombectomy. Stroke 49, 1662–1668 (2018). 29915125 10.1161/STROKEAHA.118.020567

[43] Y. Kantaros, B. V. Johnson, S. Chowdhury, D. J. Cappelleri, M. M. Zavlanos, Control of magnetic microrobot teams for temporal micromanipulation tasks. IEEE Trans. Robot. 34, 1472–1489 (2018).

[44] B. V. Johnson, S. Chowdhury, D. J. Cappelleri, Local magnetic field design and characterization for independent closed-loop control of multiple mobile microrobots. IEEE/ASME Trans. Mechatron. 25, 526–534 (2020).

[45] E. Diller, C. Pawashe, S. Floyd, M. Sitti, Assembly and disassembly of magnetic mobile micro-robots towards deterministic 2-D reconfigurable micro-systems. Int. J. Robot. Res. 30, 1667–1680 (2011).

[46] X. Fan, X. Dong, A. C. Karacakol, H. Xie, M. Sitti, Reconfigurable multifunctional ferrofluid droplet robots. Proc. Natl. Acad. Sci. U.S.A. 117, 27916–27926 (2020). 33106419 10.1073/pnas.2016388117 PMC7668164

[47] X. Dong, M. Sitti, Controlling two-dimensional collective formation and cooperative behavior of magnetic microrobot swarms. Int. J. Robot. Res. 39, 617–638 (2020).

[48] L. Yang, J. Jiang, X. Gao, Q. Wang, Q. Dou, L. Zhang, Autonomous environment-adaptive microrobot swarm navigation enabled by deep learning-based real-time distribution planning. Nat. Mach. Intell. 4, 480–493 (2022).

[49] F. Ongaro, S. Pane, S. Scheggi, S. Misra, Design of an electromagnetic setup for independent three-dimensional control of pairs of identical and nonidentical microrobots. IEEE Trans. Robot. 35, 174–183 (2019).

[50] S. Shahrokhi, J. Shi, B. Isichei, A. T. Becker, Exploiting nonslip wall contacts to position two particles using the same control input. IEEE Trans. Robot. 35, 577–588 (2019).

[51] T. Xu, C. Huang, Z. Lai, X. Wu, Independent control strategy of multiple magnetic flexible millirobots for position control and path following. IEEE Trans. Robot. 38, 2875–2887 (2022).

[52] E. Diller, J. Giltinan, M. Sitti, Independent control of multiple magnetic microrobots in three dimensions. Int. J. Robot. Res. 32, 614–631 (2013).

[53] S. Floyd, E. Diller, C. Pawashe, M. Sitti, Control methodologies for a heterogeneous group of untethered magnetic micro-robots. Int. J. Robot. Res. 30, 1553–1565 (2011).

[54] E. Diller, S. Floyd, C. Pawashe, M. Sitti, Control of multiple heterogeneous magnetic microrobots in two dimensions on nonspecialized surfaces. IEEE Trans. Robot. 28, 172–182 (2012).

[55] L. Amoudruz, P. Koumoutsakos, Independent control and path planning of microswimmers with a uniform magnetic field. Adv. Intell. Syst. 4, 2100183 (2022).

[56] L. Yang, L. Zhang, Motion control in magnetic microrobotics: From individual and multiple robots to swarms. Ann. Rev. Control Robot. Auton. Syst. 4, 509–534 (2021).

[57] M. Salehizadeh, E. D. Diller, Path planning and tracking for an underactuated two-microrobot system. IEEE Robot. Autom. Lett. 6, 2674–2681 (2021).

[58] A. Denasi, S. Misra, Independent and leader–follower control for two magnetic micro-agents. IEEE Robot. Autom. Lett. 3, 218–225 (2018).

[59] J. Davy, T. Da Veiga, G. Pittiglio, J. H. Chandler, P. Valdastri, in *2023 International Symposium on Medical Robotics (ISMR)* (IEEE, 2023), pp. 1–7.

[60] Z. Koszowska, M. Brockdorff, T. da Veiga, G. Pittiglio, P. Lloyd, T. Khan-White, R. A. Harris, J. W. Moor, J. H. Chandler, P. Valdastri, Independently actuated soft magnetic manipulators for bimanual operations in confined anatomical cavities. Adv. Intell. Syst. 6, 2300062 (2024).

[61] P. Ryan, E. Diller, in *2016 IEEE international conference on robotics and automation (ICRA)* (IEEE, 2016), pp. 1731–1736.

[62] A. W. Mahoney, J. J. Abbott, Generating rotating magnetic fields with a single permanent magnet for propulsion of untethered magnetic devices in a lumen. IEEE Trans. Robot. 30, 411–420 (2014).

[63] P. E. Hart, N. J. Nilsson, B. Raphael, A formal basis for the heuristic determination of minimum cost paths. IEEE Trans. Syst. Sci. Cybern. 4, 100–107 (1968).

[64] M. F. Hale, R. Sidhu, M. E. McAlindon, Capsule endoscopy: Current practice and future directions. World J. Gastroenterol. 20, 7752–7759 (2014). 24976712 10.3748/wjg.v20.i24.7752 PMC4069303

[65] C. McCaffrey, O. Chevalerias, C. O’Mathuna, K. Twomey, Swallowable-capsule technology. IEEE Pervasive Comput. 7, 23–29 (2008).

[66] M. R. Bennett, J. Hasty, Microfluidic devices for measuring gene network dynamics in single cells. Nat. Rev. Genet. 10, 628–638 (2009). 19668248 10.1038/nrg2625 PMC2931582

[67] H. Song, J. D. Tice, R. F. Ismagilov, A microfluidic system for controlling reaction networks in time. Angew. Chem. Int. Ed. Engl. 115, 792–796 (2003). 10.1002/anie.200390203 12596195

[68] Y. Tang, M. Li, T. Wang, X. Dong, W. Hu, M. Sitti, Wireless miniature magnetic phase-change soft actuators. Adv. Mater. 34, e2204185 (2022). 35975467 10.1002/adma.202204185 PMC7613683

[69] T. Chung, *Computational fluid dynamics* (Cambridge Univ. Press, 2010).

[70] H. Zhao, S. Coote, L. Pesavento, L. Churilov, H. M. Dewey, S. M. Davis, B. C. Campbell, Large vessel occlusion scales increase delivery to endovascular centers without excessive harm from misclassifications. Stroke 48, 568–573 (2017). 28232591 10.1161/STROKEAHA.116.016056

[71] A. K. Bonkhoff, T. Ullberg, M. Bretzner, S. Hong, M. D. Schirmer, R. W. Regenhardt, K. L. Donahue, M. J. Nardin, A. V. Dalca, A.-K. Giese, Deep profiling of multiple ischemic lesions in a large, multi-center cohort: Frequency, spatial distribution, and associations to clinical characteristics. Front. Neurosci. 16, 994458 (2022). 36090258 10.3389/fnins.2022.994458 PMC9453031

[72] J. L. Brisman, J. K. Song, D. W. Newell, Cerebral aneurysms. N. Engl. J. Med. 355, 928–939 (2006). 16943405 10.1056/NEJMra052760

[73] M. N. Shah, S. E. Smith, D. L. Dierker, J. P. Herbert, T. S. Coalson, B. S. Bruck, G. J. Zipfel, D. C. Van Essen, R. G. Dacey, The relationship of cortical folding and brain arteriovenous malformations. Neurovasc. Imaging 2, 1–10 (2016). 10.1186/s40809-016-0024-3 PMC5167380 28009020

[74] R. Willinsky, P. Lasjaunias, K. Terbrugge, P. Burrows, Multiple cerebral arteriovenous malformations (AVMs) Review of our experience from 203 patients with cerebral vascular lesions. Neuroradiology 32, 207–210 (1990). 2215905 10.1007/BF00589113

[75] K.-P. Stein, I. Wanke, N. Oezkan, Y. Zhu, I. E. Sandalcioglu, M. Forsting, U. Sure, Multiple cerebral arterio-venous malformations: Impact of multiplicity and hemodynamics on treatment strategies. Acta Neurochir. 158, 2399–2407 (2016). 27766428 10.1007/s00701-016-2989-8

[76] A. Rahmanian, M. R. Farrokhi, E. A. Alibai, M. S. Masoudi, Multiple intracranial dural arteriovenous fistula. J. Res. Med. Sci. 18, 360–362 (2013). 24124437 PMC3793385

[77] J. Gould, A. McDonald, Breaking down brain cancer. Nature 561, S40–S41 (2018). 30258156 10.1038/d41586-018-06704-7

[78] M. F. Dempsey, B. R. Condon, D. M. Hadley, Measurement of tumor “size” in recurrent malignant glioma: 1D, 2D, or 3D? AJNR Am. J. Neuroradiol. 26, 770–776 (2005). 15814919 PMC7977136

