# Supplementary material for: Heterogeneous multiple soft millirobots in three-dimensional lumens
Source: Sci Adv. 2024 Nov 6;10(45):eadq1951. doi: 10.1126/sciadv.adq1951 (PMC11540014; doi:10.1126/sciadv.adq1951)
Supplement: Supplementary file 1 — Figs. S1 to S24 Table S1 Legends for movies S1 to S5 References [file sciadv.adq1951_sm.pdf]

Science Advances
AAAS

Supplementary Materials for

Heterogeneous multiple soft millirobots in three-dimensional lumens

Chunxiang Wang *et al.*

Corresponding author: Tianlu Wang, tianluw@hawaii.edu; Metin Sitti, sitti@is.mpg.de

Sci. Adv. **10**, eadq1951 (2024)
DOI: 10.1126/sciadv.adq1951

The PDF file includes:

Figs. S1 to S24
Table S1
Legends for movies S1 to S5
References

Other Supplementary Material for this manuscript includes the following:

Movies S1 to S5

Supplementary Figures

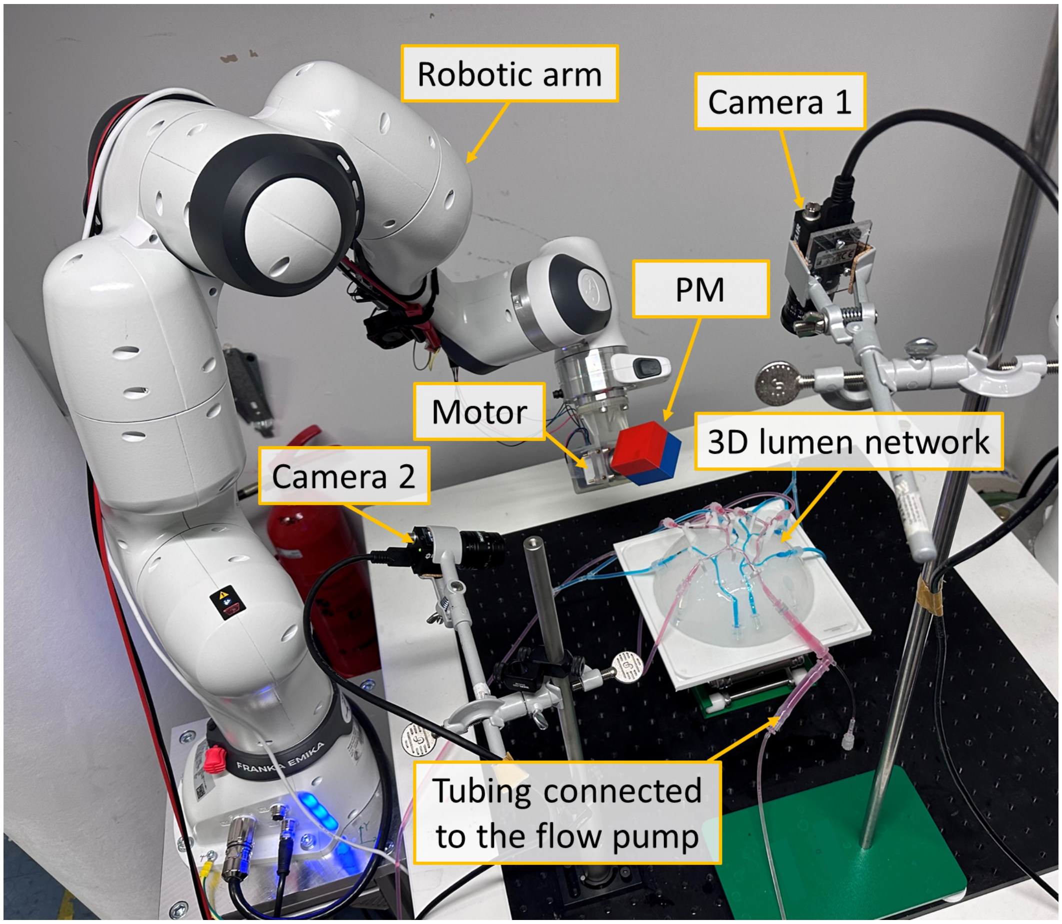

Fig. S1. Robotic system for multi-robot deployment.

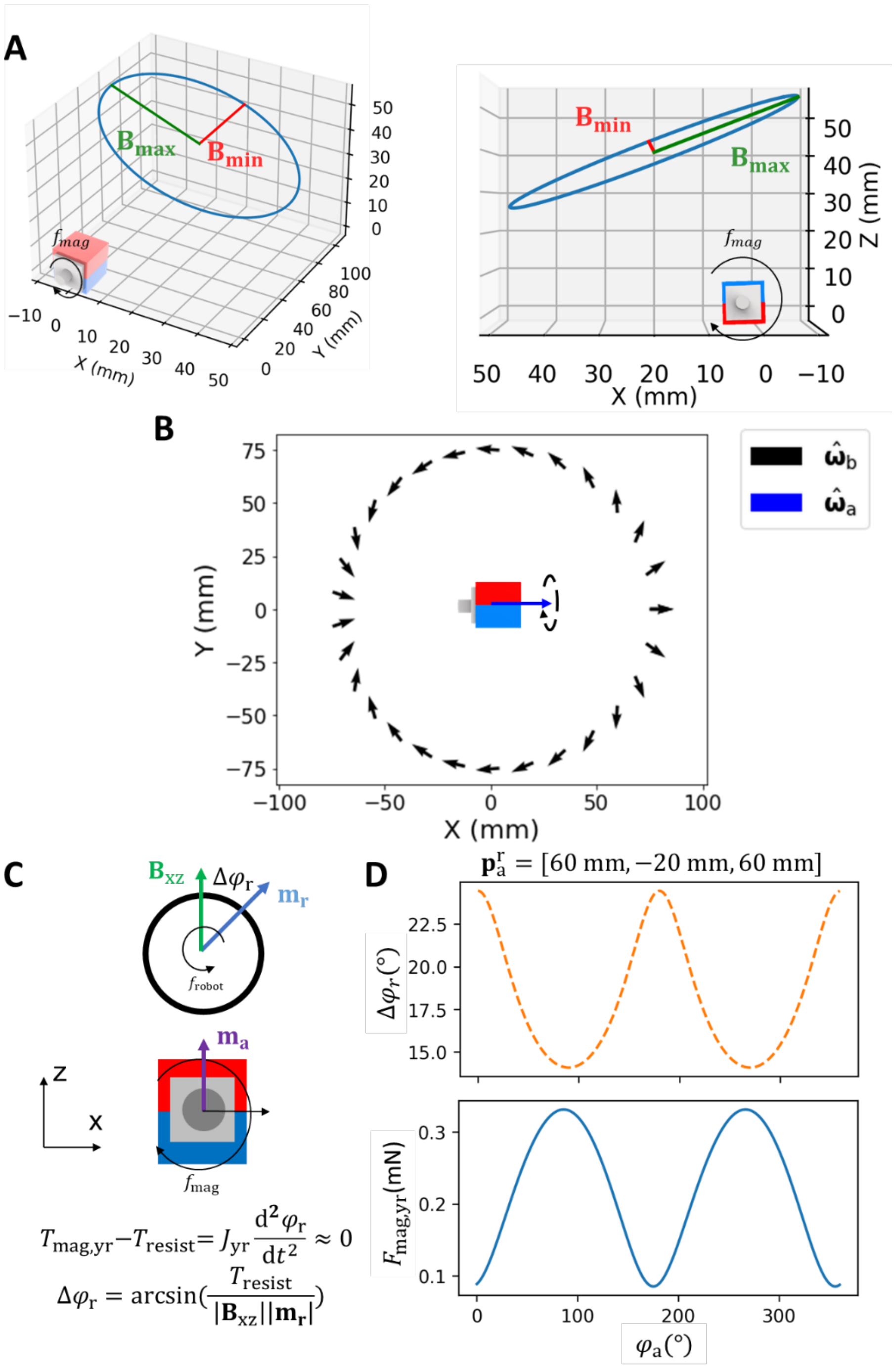

Fig. S2. Magnetic field and force generated by a rotating permanent magnet. A. Magnetic field generated by the rotating permanent magnet (PM). The generated magnetic field by a PM rotates around a constant axis, and its field magnitude is described by an ellipse with the maximum and minimum magnetic fields $\mathbf{B}_{\mathbf{max}}$ and $\mathbf{B}_{\mathbf{min}}$. **B.** Relationship between the rotating axis of the PM, $\widehat{\boldsymbol{\omega}}_a$, and the rotating axis of the rotating magnetic field, $\widehat{\boldsymbol{\omega}}_b$. **C.** Calculation of the angle difference $\Delta\varphi_r$ between the robot magnetic moment $\mathbf{m}_r$ and the rotating magnetic field projected onto the $x_r - z_r$ plane of the robot-body-attached coordinate $\mathbf{B}_{xz}$. **D.** Variation of the magnetic force along the y_r-axis, $F_{mag,yr}$, and $\Delta\varphi_r$ with the PM rotation angle φ_a.

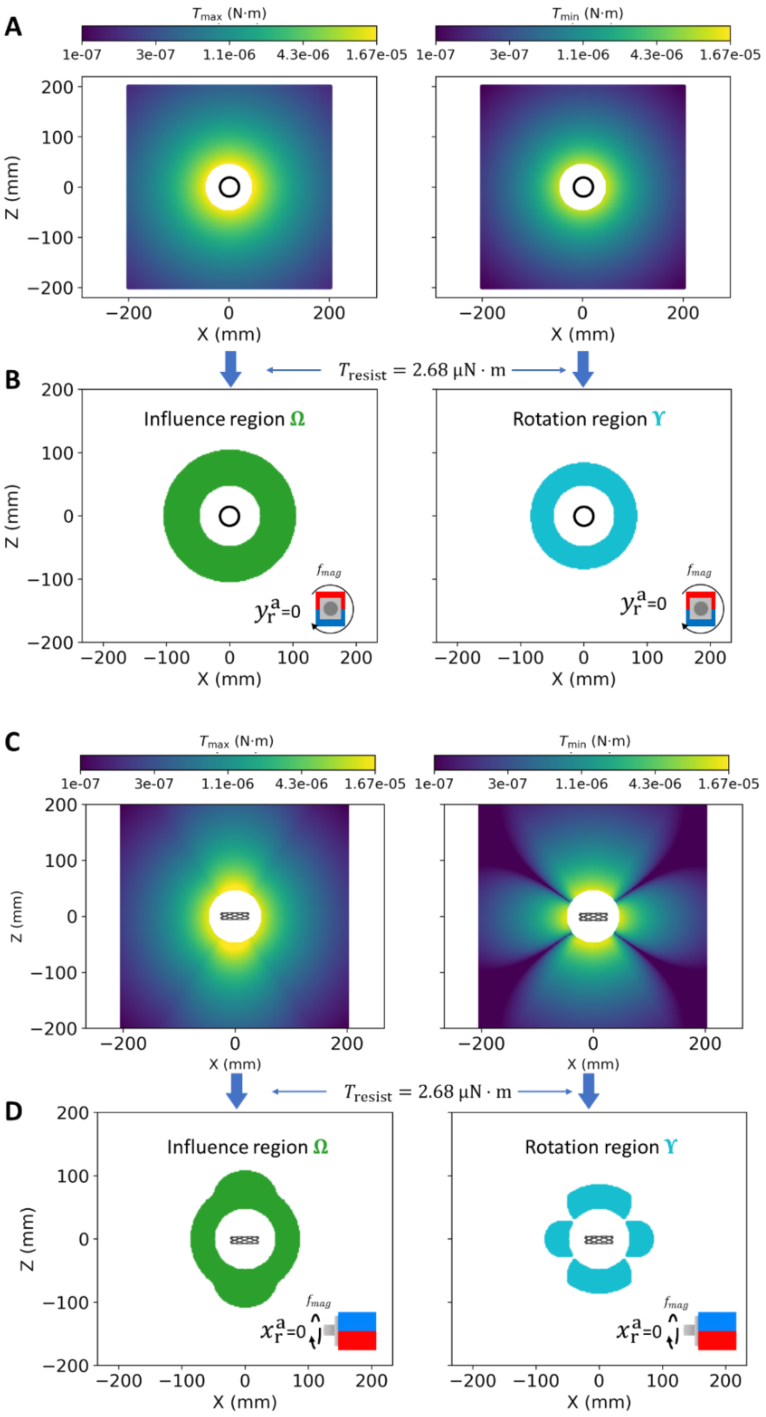

Fig. S3. Influence and rotation regions for robot rotation. A. Distribution of the maximum magnetic torque T_{max} and minimum magnetic torque T_{min} on the plane y_r^a=0. **B.** Determination of the influence region $\boldsymbol{\Omega}$ and rotation region $\boldsymbol{\Upsilon}$. Inside $\boldsymbol{\Omega}$, T_{max} surpasses the resistance torque T_{resist}, while T_{min} exceeds T_{resist} inside $\boldsymbol{\Upsilon}$. **C.** Distribution of T_{max} and T_{min} on the plane x_r^a=0. **D.** Shapes of the $\boldsymbol{\Omega}$ and $\boldsymbol{\Upsilon}$ on the plane x_r^a=0. Due to the serious misalignment between the robot and PM rotation axes, the robot cannot rotate continuously even if the PM is quite close to it in the corner region inside $\boldsymbol{\Omega}$ while outside $\boldsymbol{\Upsilon}$.

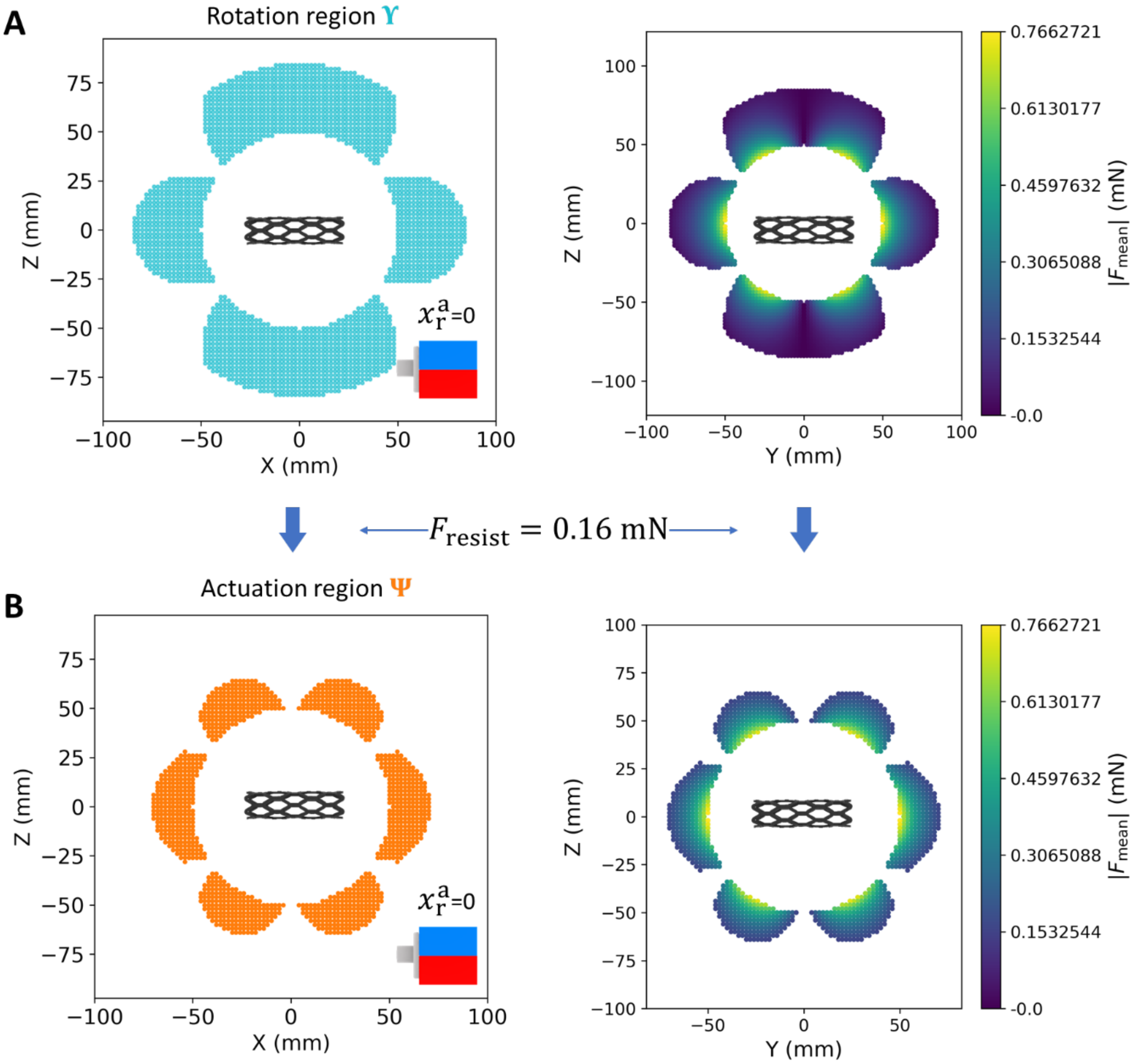

Fig. S4. Actuation region for robot translation. A. Distribution of the mean magnetic force F_{mean} inside $\mathbf{\Upsilon}$. The continuously rotation of the robot inside $\mathbf{\Upsilon}$ is a prerequisite for translation. **B.** Determination of the actuation region $\mathbf{\Psi}$. Inside $\mathbf{\Psi}$, T_{min} exceeds T_{resist}, and F_{mean} overpasses the resistance force F_{resist}, enabling the continuous rotation and translation of the robot.

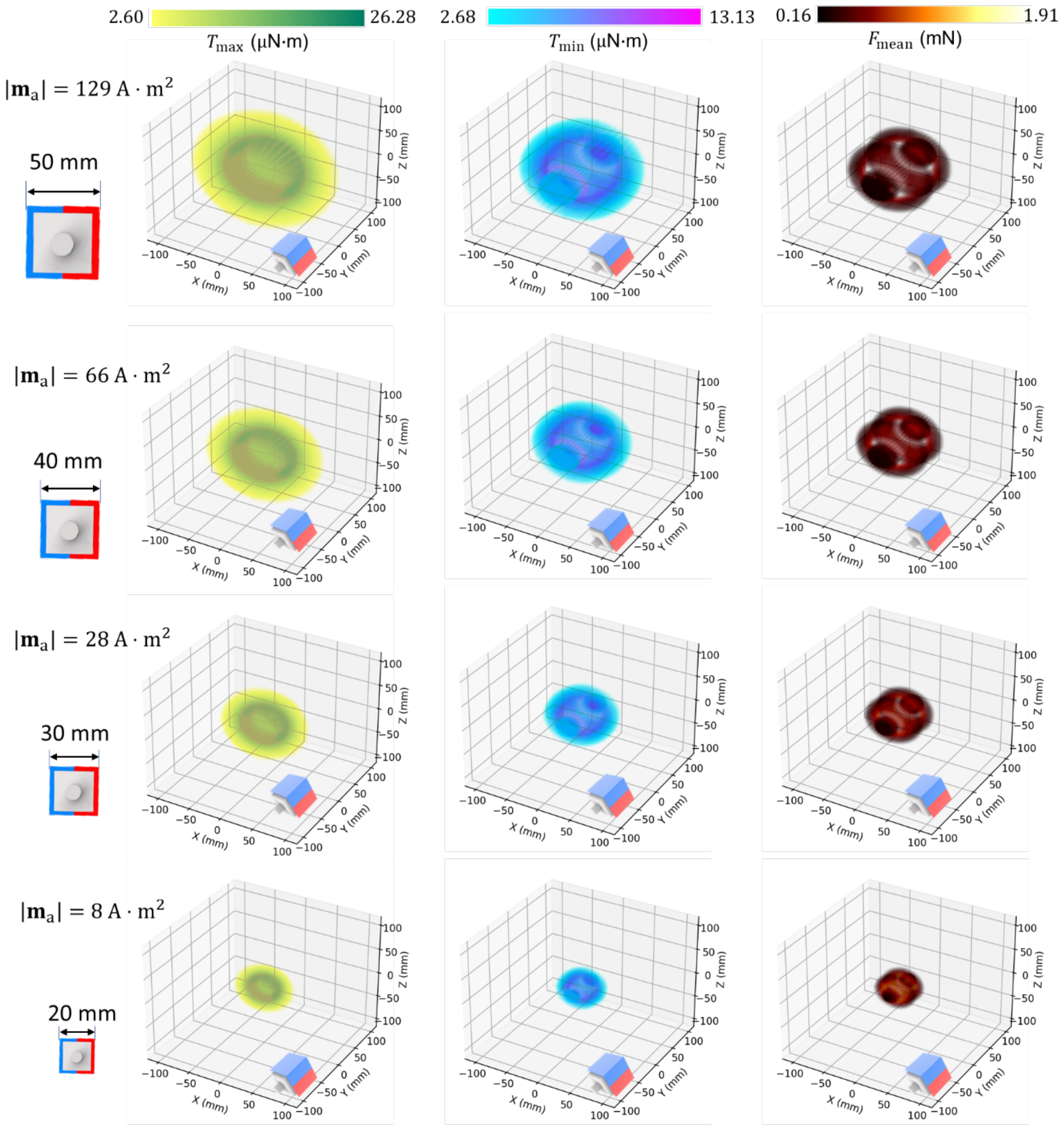

Fig. S5. Influence of magnet size on influence, rotation, and actuation regions.

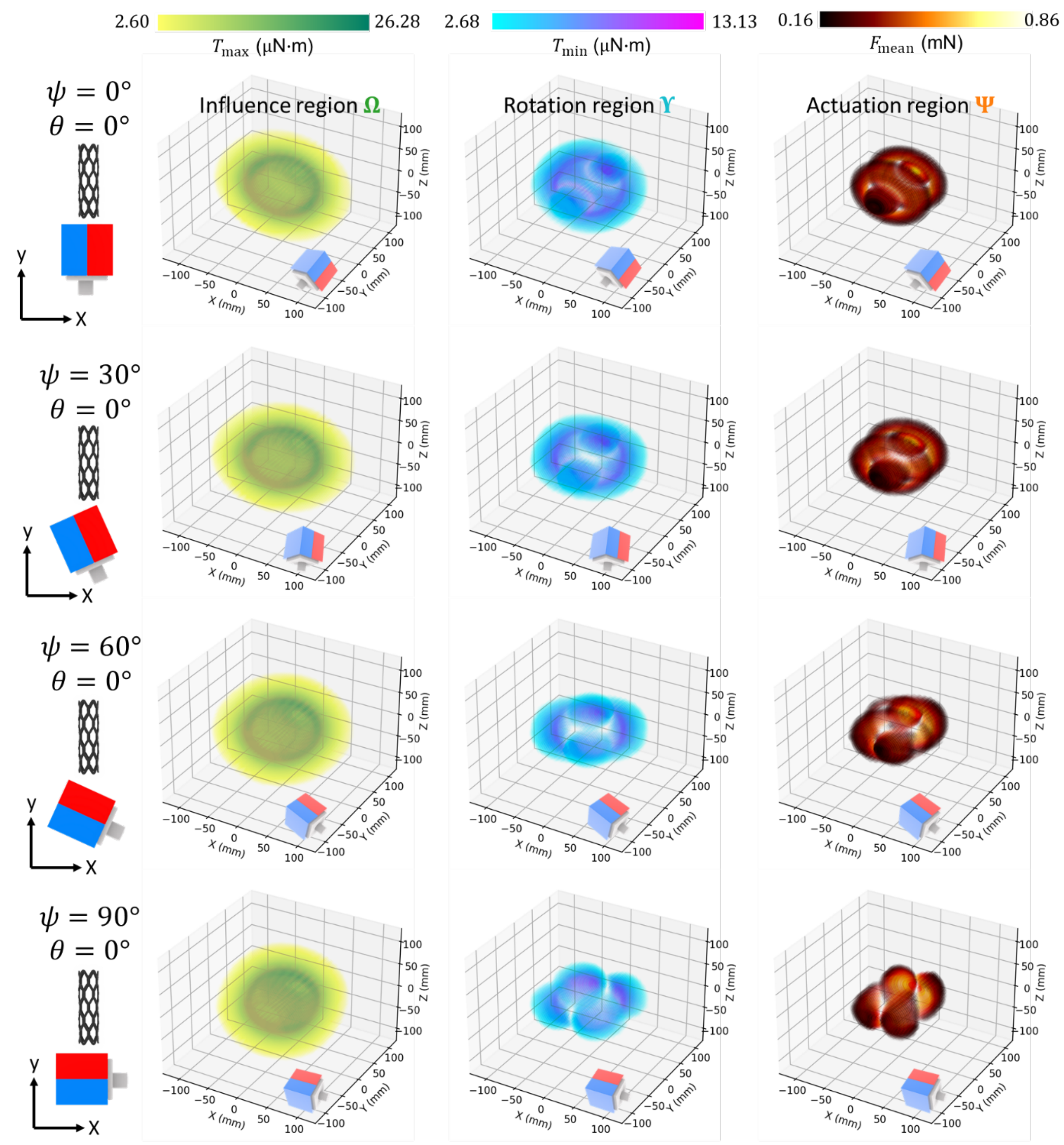

Fig. S6. Influence of magnet orientation on influence, rotation, and actuation regions.

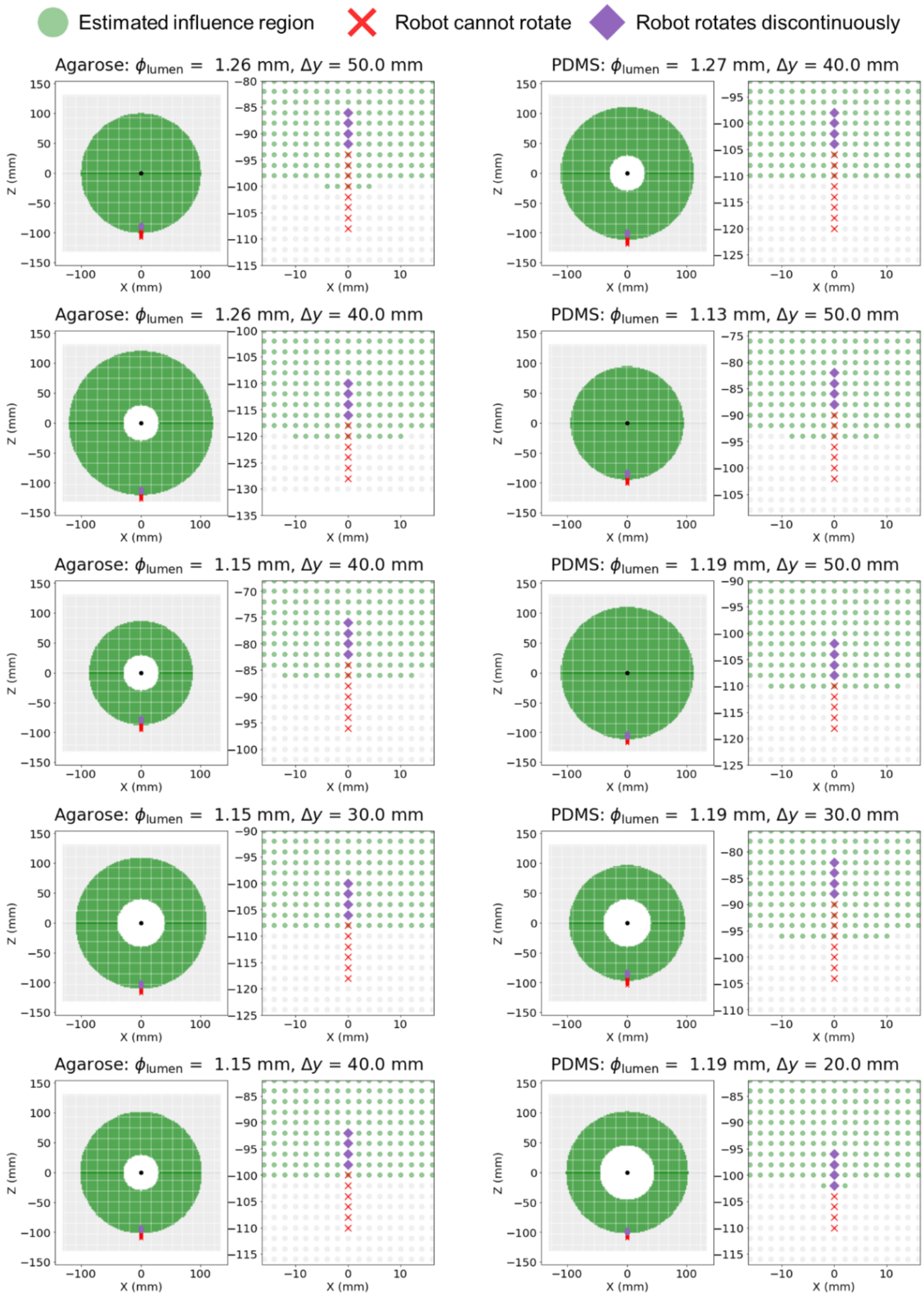

Fig. S7. Experiment validation of the influence region. For the validation of the influence region, the resistance torque is firstly estimated, and then the influence region is computed. Subsequently, the PM relocates to Δy along the y_{r} −axis and approaches the robot along the z_{r} −axis, during which the robot rotation is observed and compared with the computed region.

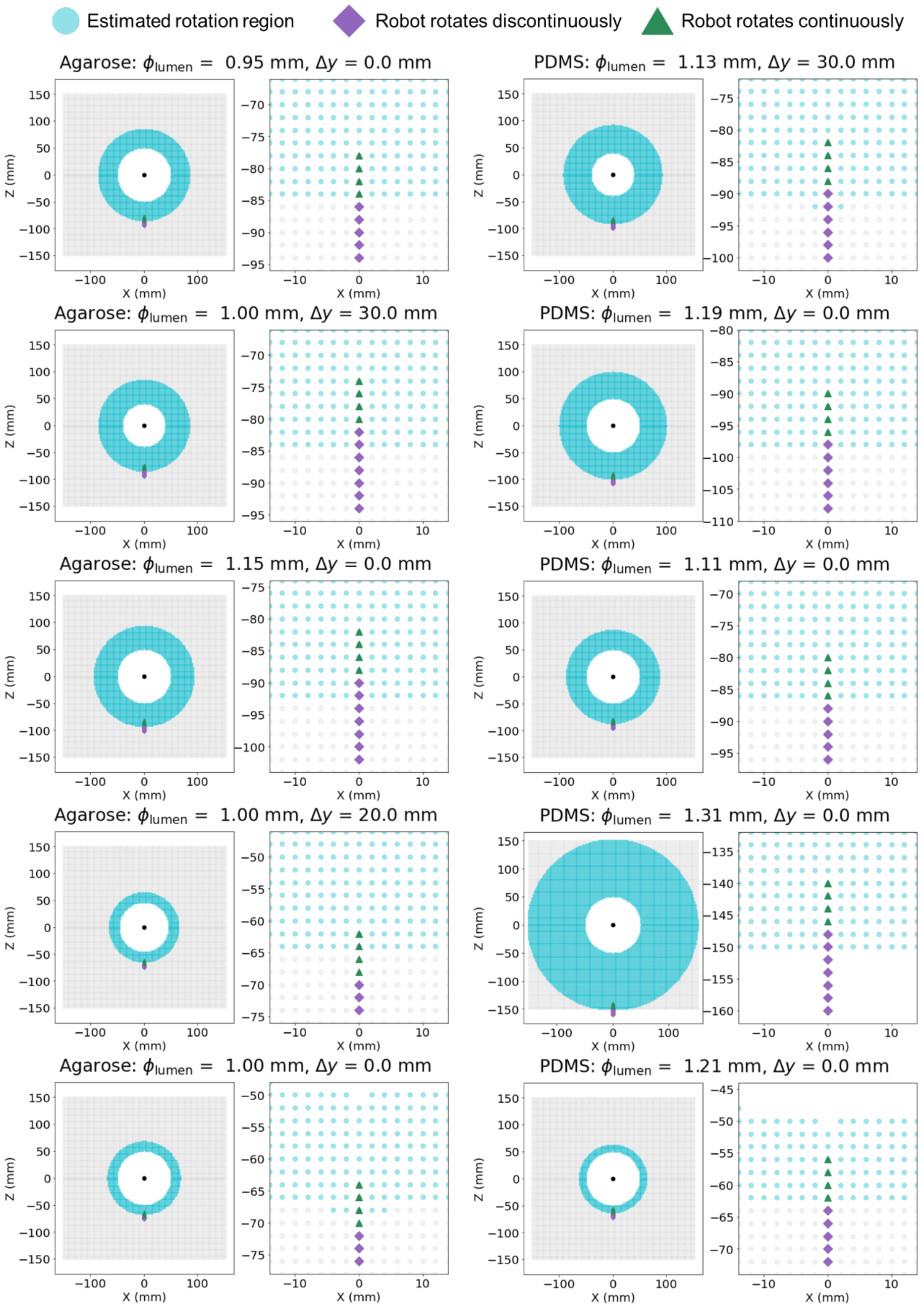

Fig. S8. Experiment validation of the rotation region. For the validation of the rotation region, the resistance torque is firstly estimated, followed by computation of the rotation region. Subsequently, the PM relocates to Δy along the y_{r} −axis and approaches the robot along the z_{r} −axis, during which the robot rotation is observed and compared with the computed region.

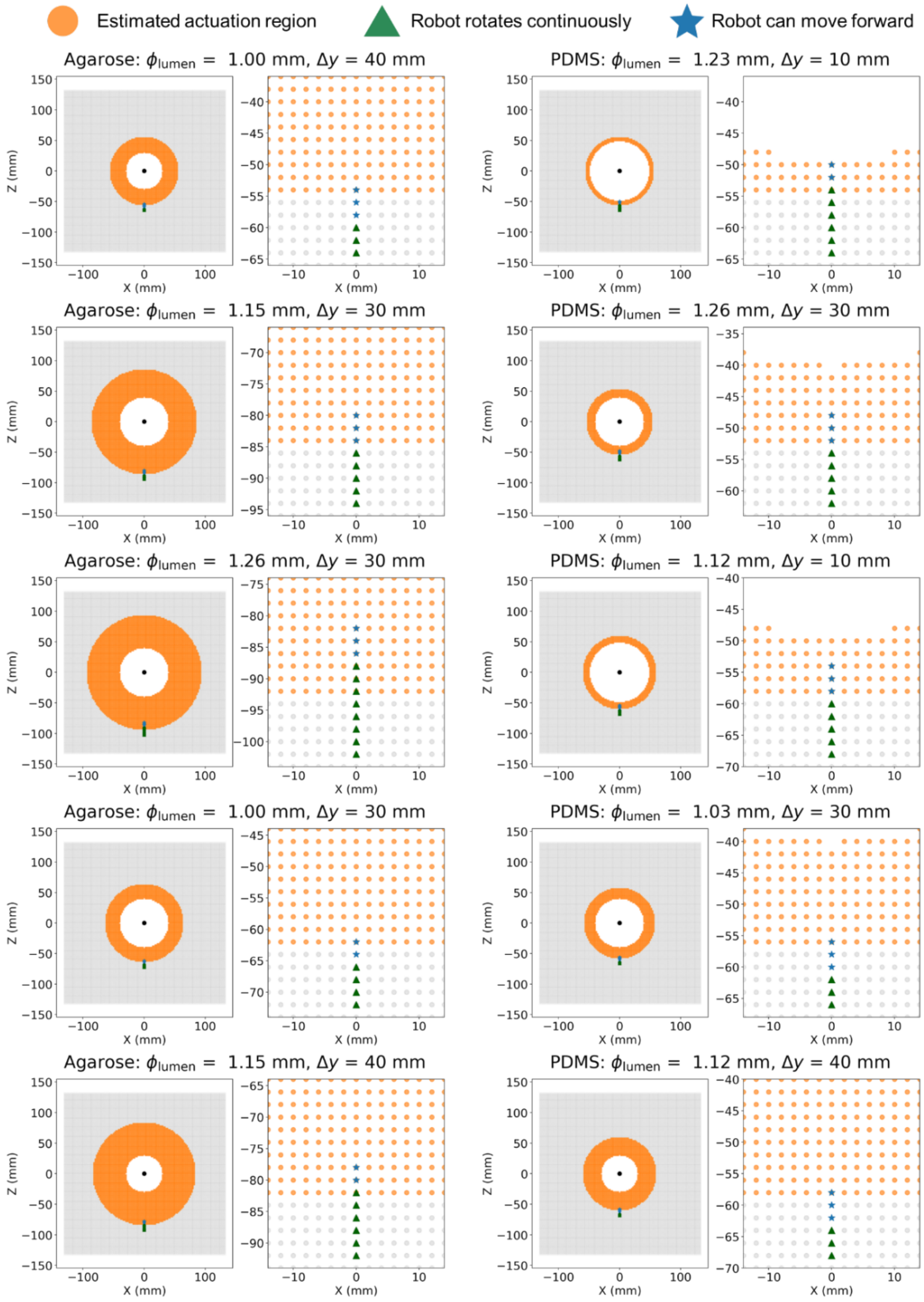

Fig. S9. Experiment validation of the actuation region. For the validation of the actuation region, the resistance torque and force are firstly estimated, and then the actuation region is computed. Subsequently, the PM relocates to Δy along the y_r −axis and approaches the robot along the z_r −axis, during which the robot rotation is observed and compared with the computed region.

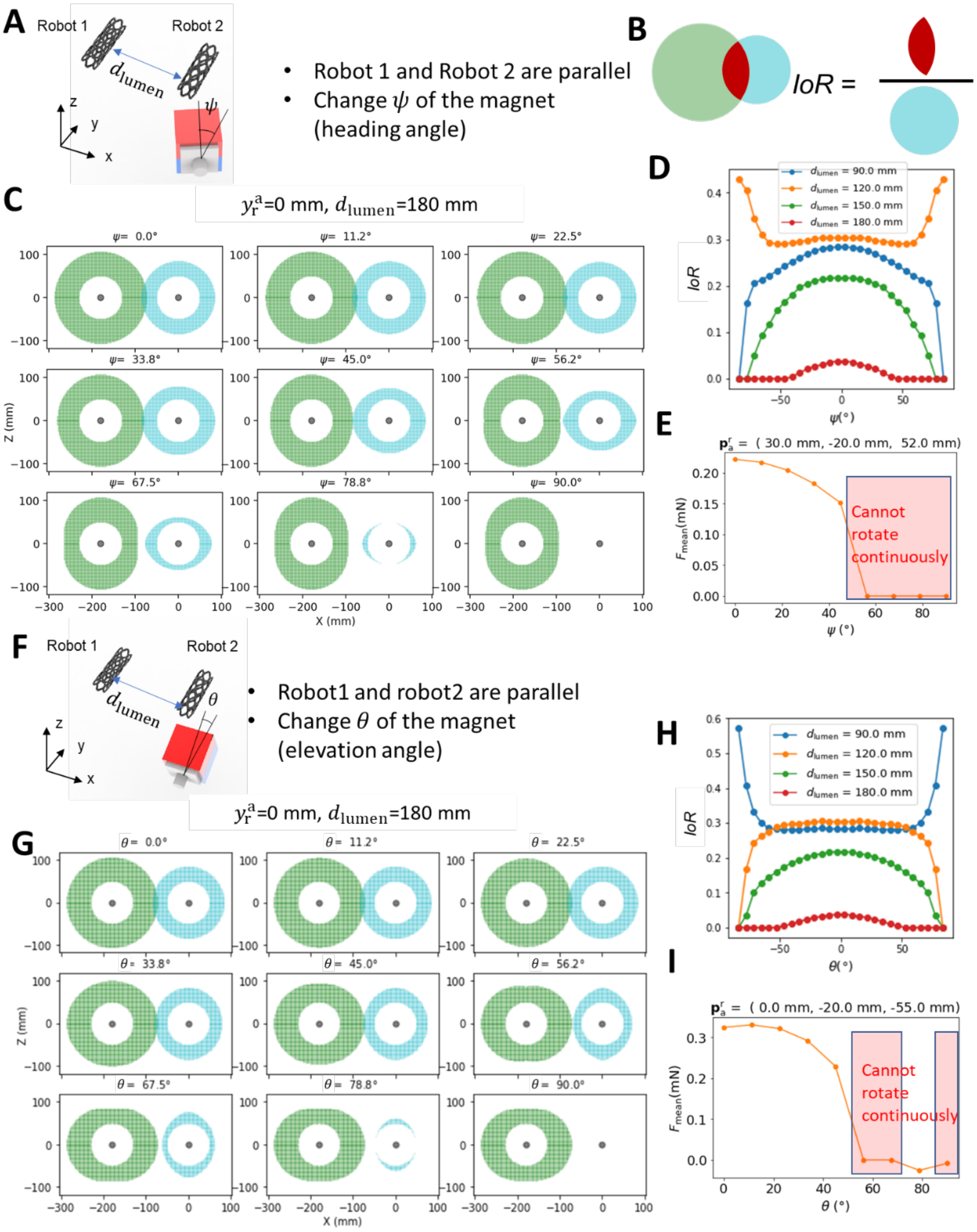

Fig. S10. Influence of magnet orientation on independent robot control. A. Schematic of investigating the influence of the PM heading angle ψ on the independent control of two parallel robots. **B.** Definition of Intersection over Rotation region (IoR). IoR is the ratio of the intersection area of $\mathbf{\Omega}$ and $\mathbf{\Upsilon}$ to the area of $\mathbf{\Upsilon}$. **C.** Variation of $\mathbf{\Omega}$ and $\mathbf{\Upsilon}$ with ψ at the plane y_r^a=0. **D.** Variation of IoR with ψ and lumen distance d_{lumen} at the plane y_r^a=0. **E.** Variation of F_{mean} with ψ. **F.** Schematic of investigating the influence of the PM elevation angle θ on the independent control of of two parallel robots. **G.** Variation of $\mathbf{\Omega}$ and $\mathbf{\Upsilon}$ with θ at the plane y_r^a=0. **H.** Variation of IoR with θ and lumen distance d_{lumen} at the plane y_r^a=0. **I.** Variation of F_{mean} with θ.

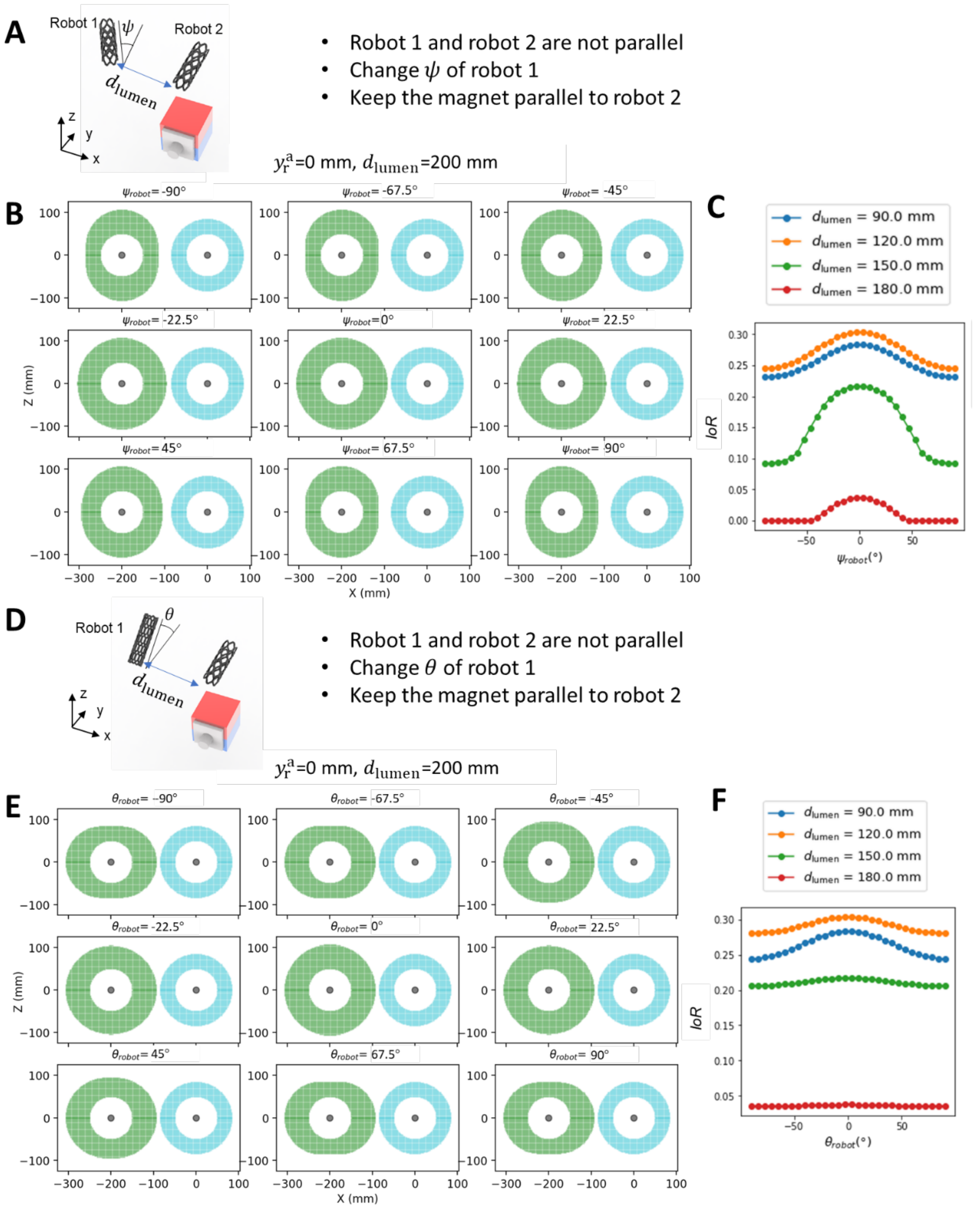

Fig. S11. Influence of robot orientation on independent robot control. A. Schematic of investigating the influence of the robot heading angle ψ on the independent control. **B.** Variation of $\mathbf{\Omega}$ and $\mathbf{\Upsilon}$ with ψ at the plane y_r^a=0. **C.** Variation of IoR with ψ and lumen distance d_{lumen} at the plane y_r^a=0. The magnetic force for robot 2 remains fixed. **D.** Schematic of investigating the influence of the robot elevation angle θ on the independent control. **E.** Variation of $\mathbf{\Omega}$ and $\mathbf{\Upsilon}$ with θ at the plane y_r^a=0. **F.** Variation of IoR with θ and lumen distance d_{lumen} at the plane y_r^a=0. The magnetic force for robot 2 remains fixed.

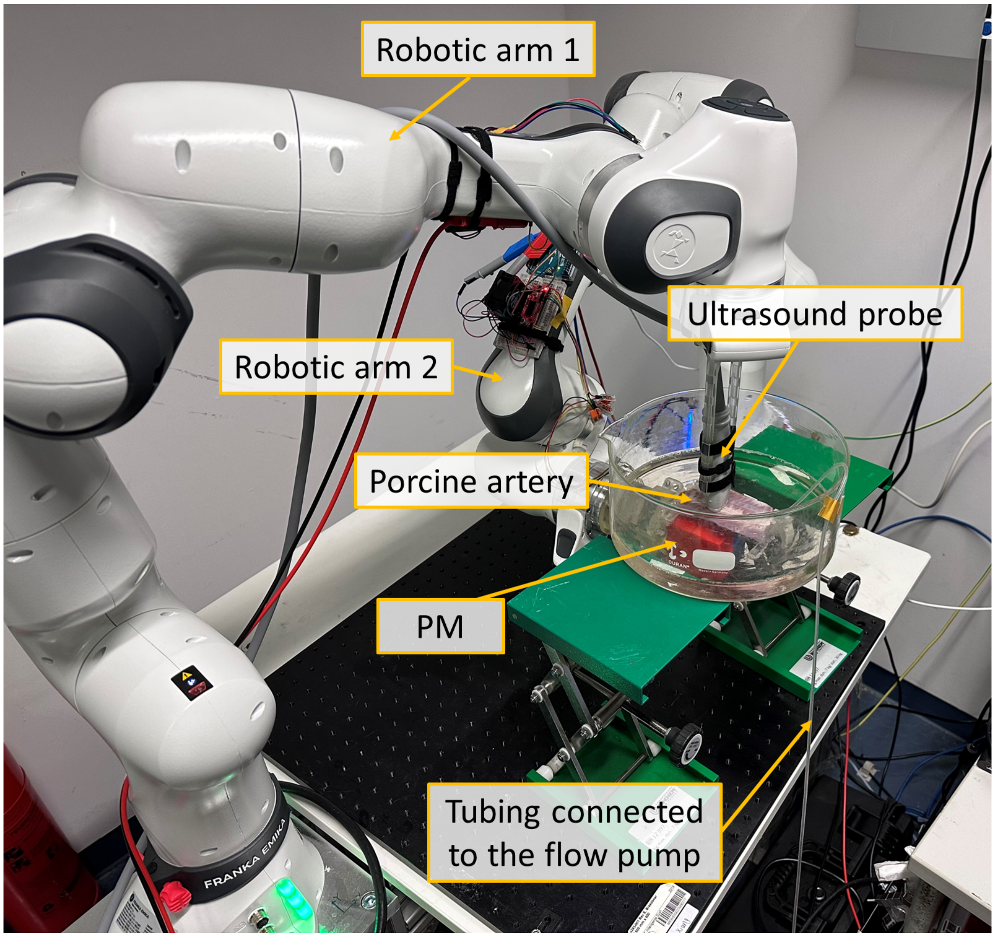

Fig. S12. Robotic system for robot deployment within porcine arteries.

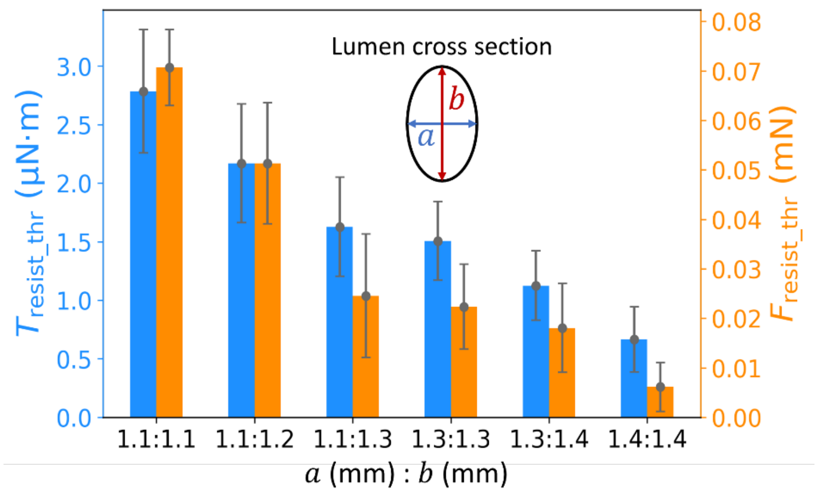

Fig. S13. Influence of lumen roundness on the resistance. The resistive torque and force thresholds in an elliptical lumen fall between those for circular lumens with diameters equal to the long and short axes of the elliptical lumen. The straight lumens were prepared with 1.0 wt% agarose gel. Error bars denote the standard deviation of measurements from one sample using four robots.

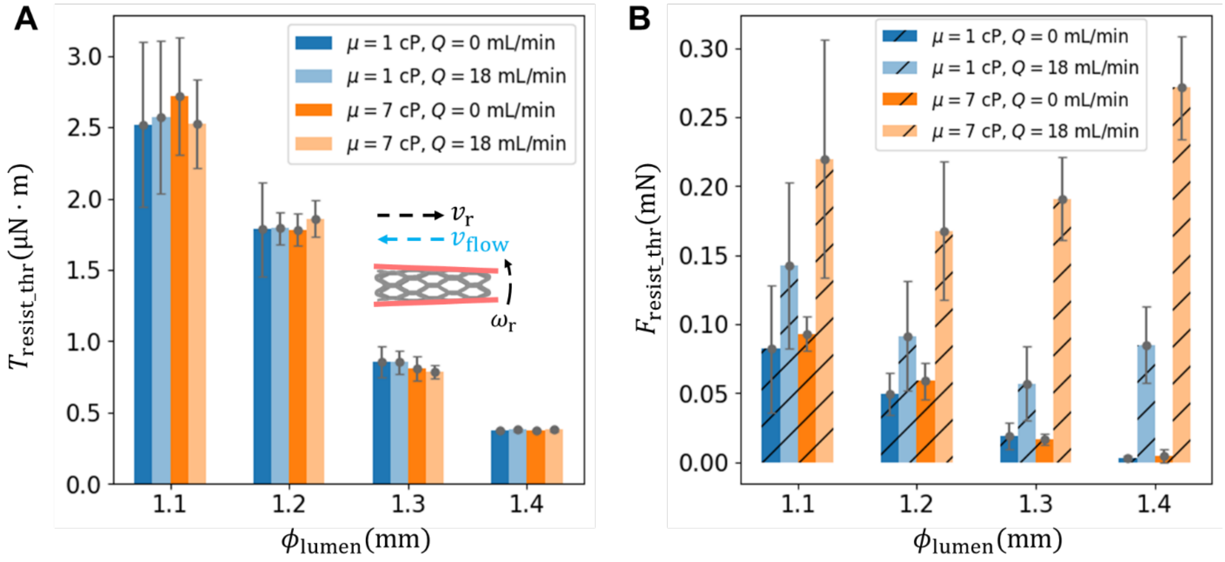

Fig. S14. Influence of fluid viscosity and flow speed on resistive thresholds. A. Effect on the resistive torque threshold (T_{resist_thr}). Increasing fluid viscosity and flow speed have slight effect on T_{resist_thr}. **B.** Effect on the resistive force threshold (F_{resist_thr}). Higher viscosity (μ) and flow rate (Q) increase fluid drag (F_{fluid}), resulting in a larger F_{resist_thr} as the robot moves against the flow. Fluid flow is modeled as Poiseuille flow, with velocity profile $u(r) = u_{max}(1 - 4r^2/\phi_{lumen}^2)$, where r is the distance from the lumen centerline, and u_{max} is the flow speed along the centerline. Fluid drag is modelled as $F_{fluid} = 0.5\rho u^2 c_d A$, where ρ is the mass density of the fluid, u is the flow velocity relative to the robot, A is the reference area, and c_d is the drag coefficient relating to the robot geometry and viscosity (*69*). Increasing fluid viscosity lead to a larger c_d and consequently enhanced F_{fluid}. The influence of ϕ_{lumen} on F_{resist_thr} was also studied. When the robot surface tightly contacts the lumen wall, decreasing ϕ_{lumen} leads to a larger u under a constant Q, further raising F_{fluid}. In large lumens ϕ_{lumen} = 1.4 mm, the fluid filling the gap between the robot surface and lumen wall results in fluid lubrication and higher u around the robot outer surface, further enhancing F_{fluid}. For robot deployment against the flow, the estimation of F_{resist_thr} is conducted at various ϕ_{lumen}, and $F_{resist\_thr}(\phi_{lumen})$ is fitted via linear interpolation, which is further scaled up to compute the actuation region ($\boldsymbol{\Psi}$). All experiments were conducted in a tapering lumen inside (1.0 wt% agarose gel) with a constant Q. Error bars denote the standard deviation of measurements from one sample using four robots.

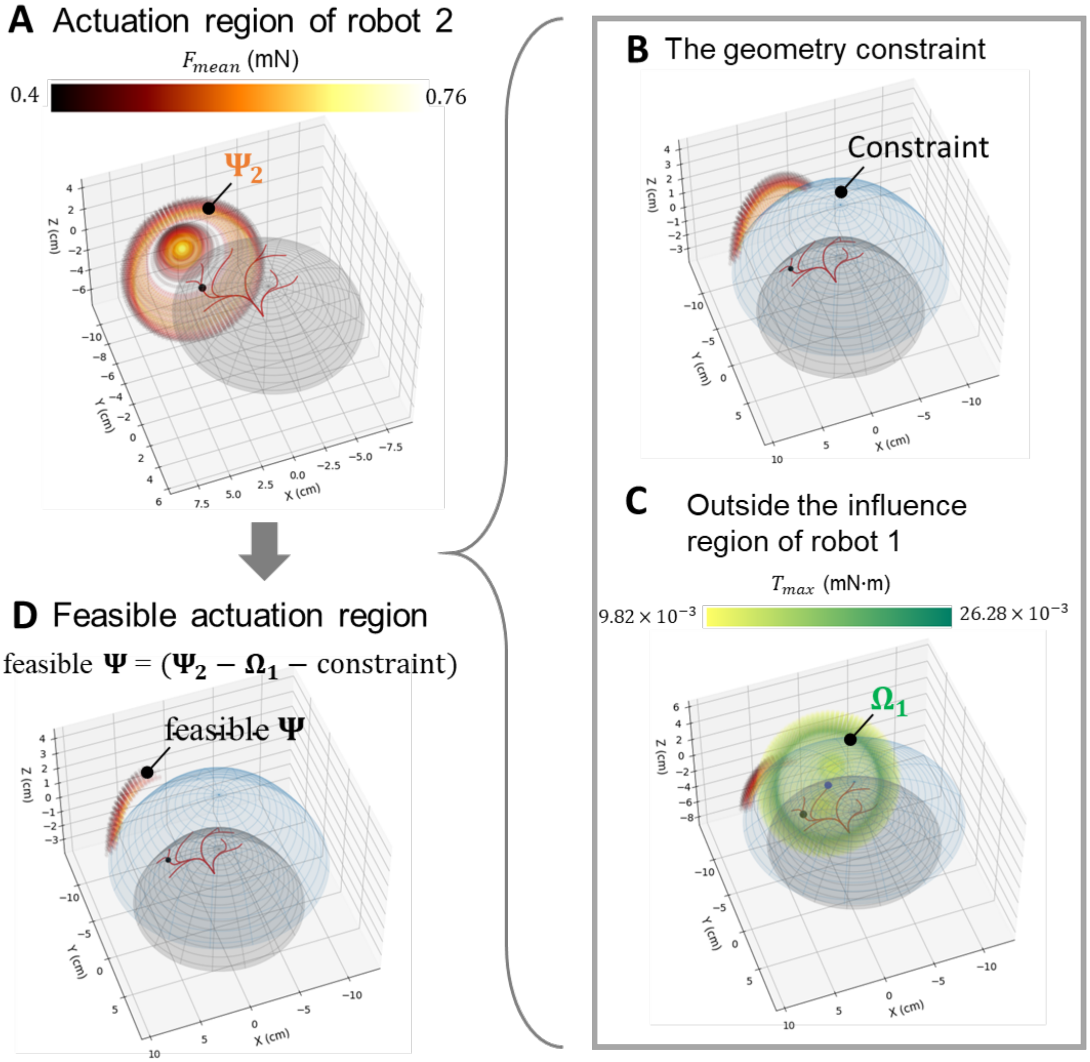

Fig. S15. Feasible actuation region for multi-robot deployment. A. Actuation region for the robot. **B.** Actuation region subtracted by the geometry constraint to avoid the collision between the PM and the phantom. **C.** Actuation region subtracted by the geometry constraint and influence region of the deployed robot. **D.** Definition of the feasible actuation region.

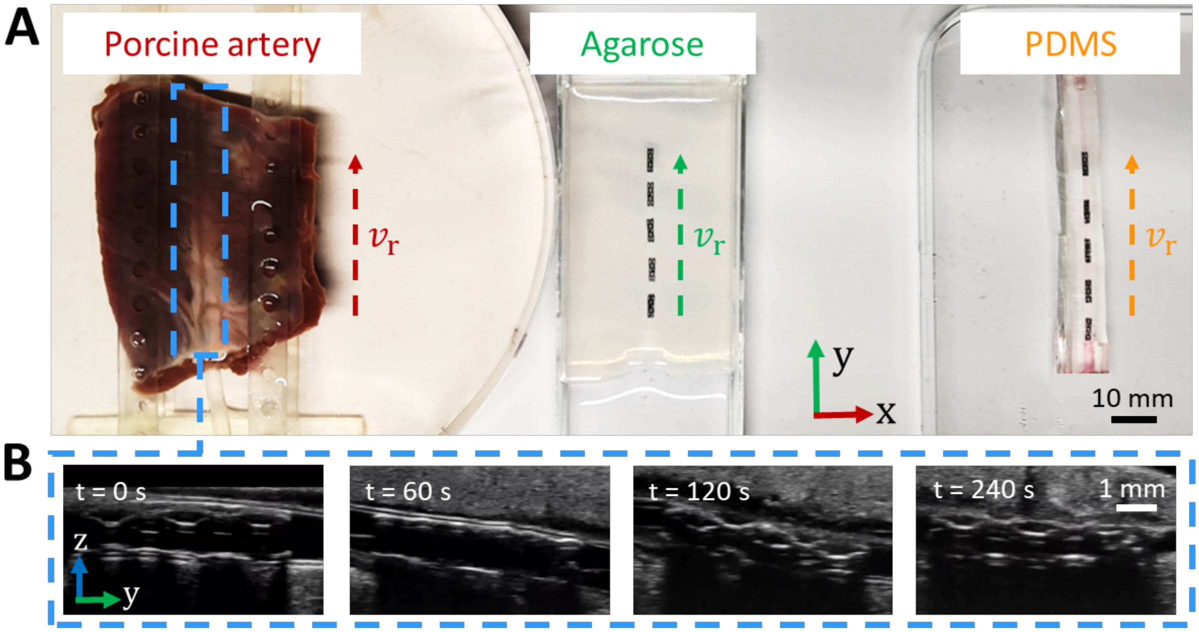

Fig. S16. Deployment of three robots into ex-vivo porcine arteries and synthetic lumens. A. Sequential deployment of three robots into the porcine artery, a lumen of agarose gel, and a lumen of PDMS. **B.** Monitoring the robot deployment inside the porcine artery under the ultrasound imaging (Vevo 3100, FUJIFILM Visualsonics, Inc.). The deployment process was also demonstrated in **Movie. S3.**

Path Planning Algorithm

Input:

1. $\mathcal{L} = \{\mathbf{L}_1, \mathbf{L}_2, \ldots, \mathbf{L}_N\}$, $\mathbf{L}_i = \{[\mathbf{p}_1, \mathbf{p}_2, \ldots, \mathbf{p}_M], [\phi_1, \phi_2, \ldots, \phi_M]\}$

▷ $\mathcal{L}$ includes the lumen information L of N target lumens.

▷ L_i includes M points $\mathbf{p} = (\mathrm{x}, \mathrm{y}, \mathrm{z})$ and the corresponding lumen diameter ϕ.

2. $\mathcal{R} = \{\mathbf{R}_1, \mathbf{R}_2, \ldots, \mathbf{R}_N\}$, $\mathbf{R}_i = \{T_{\mathrm{resist\_thr}}(\phi), F_{\mathrm{resist\_thr}}(\phi)\}$

▷ $\mathcal{R}$ includes the robot information $\mathbf{R}$ of N robots

3. $\mathcal{M} = \{A_1, A_2, \ldots, A_Q\}$

▷ $\mathcal{M}$ includes the magnet moment amplitude of Q magnets, and $A_{i-1} > A_i$.

4. $\mathbf{G}$ ▷ Feasible region for placing the magnet to avoid collision with the environment.

Output:

1. $\mathcal{D} = [\mathbf{d}_1, \mathbf{d}_2, \ldots, \mathbf{d}_N]$, $\mathbf{d}_i = \{\mathbf{L}_a, \mathbf{R}_b, \mathbf{P}_i, \mathbf{M}_i\}$

▷ $\mathbf{d}_i$ is the i-th deployment information, where the b-th robot is deployed to the a-th lumen with the c-th magnet, following the magnet path $\boldsymbol{P}_i$.

2. $\mathbf{P}_i = [\mathbf{p}_1, \mathbf{p}_2, \ldots, \mathbf{p}_M]$, $\mathbf{M}_i = [m_1, m_2, \ldots, m_M]$, $m_i \epsilon\ \mathcal{M}$

▷ $\mathbf{P}_i$, $\mathbf{M}_i$ includes the magnet position $\mathbf{p}$ and the corresponding magnet

Procedure:

```
FOR each path_order in Permutation(N)                    ▷ path_order = [1,2] or [2,1] if N=2
  FOR each robot_order in Permutation(N)
    FOR i = 1 to i = N                                    ▷ N lumens
      FOR j = 1 to j = M                                  ▷ M points in the lumen
        FOR q = 1 to q = Q                                ▷ Q magnets to choose
          Ψ_feasible = (Ψ_i − Ω_{k=1:i−1}) ∩ G
          IF Ψ_feasible exists THEN
            P_i[j] = max_p(F_mean(p) − α_1|p − P_i[j − 1]|z − α_2 l_normal), p ∈ Ψ_feasible
            M_i[j] = A_q            ▷look for the point p with the maximum
            flag = True             magnetic force and path continuity
          ELSE:                     ▷ l_normal is the orthogonal distance between p and
            flag = False            robot point on the ellipsoid surface
          ENDIF
        ENDFOR
        IF flag is False THEN
          break
        ENDIF
      ENDFOR
      IF flag is True THEN
        d_i = {L_path_order[i], R_robot_order[i], P_i, M_i}
      ELSE:
        break
      ENDIF
    ENDFOR
    IF flag is True THEN                  ▷ end the loop if the current
      end the planning process            deployment order is feasible
    ENDIF
  ENDFOR
ENDFOR
```

Fig. S17. Pseudo-code representation of the path planning algorithm.

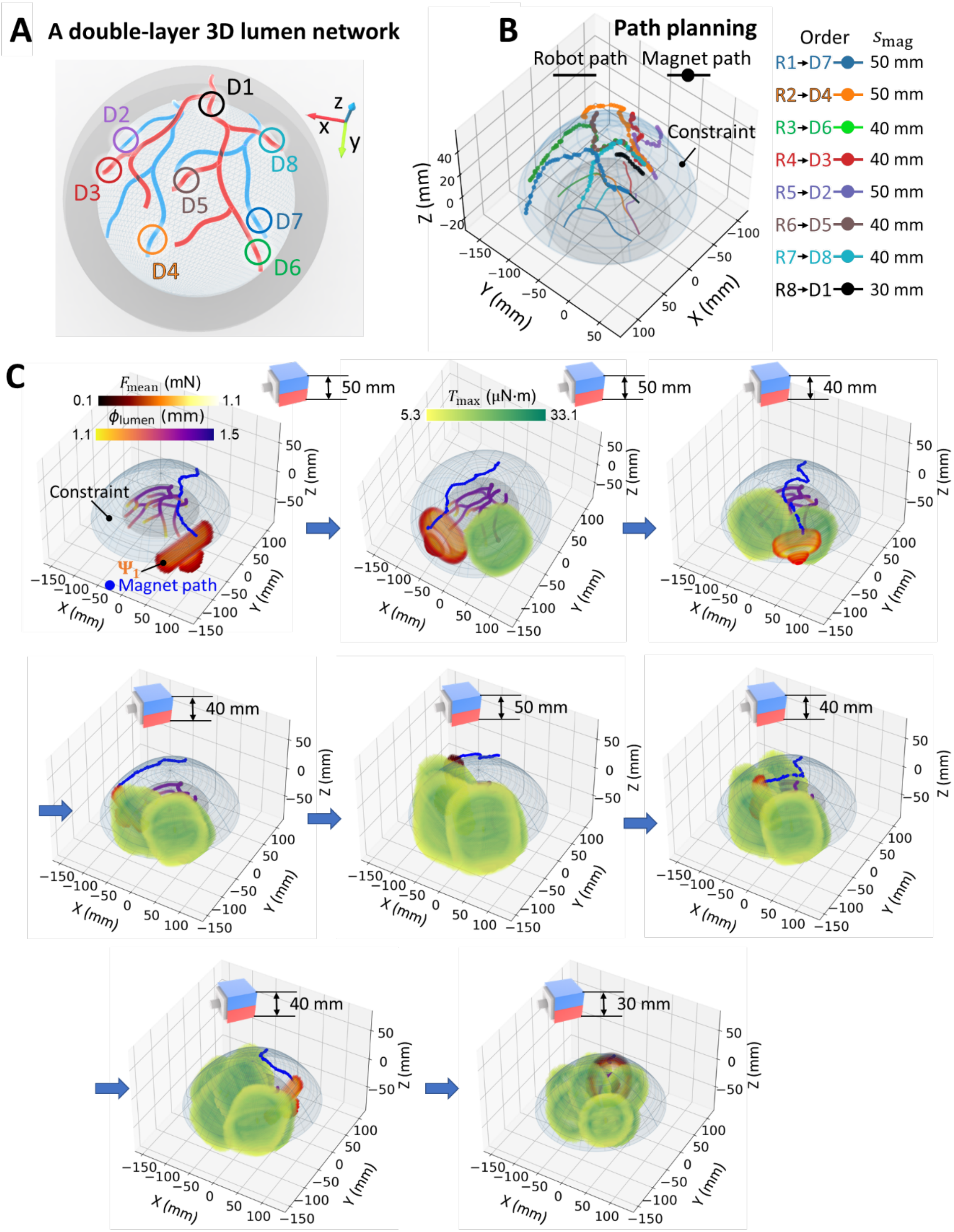

Fig. S18. Path planning of eight robots within a double-layer 3D lumen network. A. Geometry of the double-layer 3D lumen network with targeted deployment destinations D1-D8 on two layers. **B.** Path planning result for the multi-robot deployment. **C.** Magnet paths for the deployment of robot R1-8. The magnet path, **Ω**s of deployed robots, and **Ψ** of the actuated robot at the destination are illustrated.

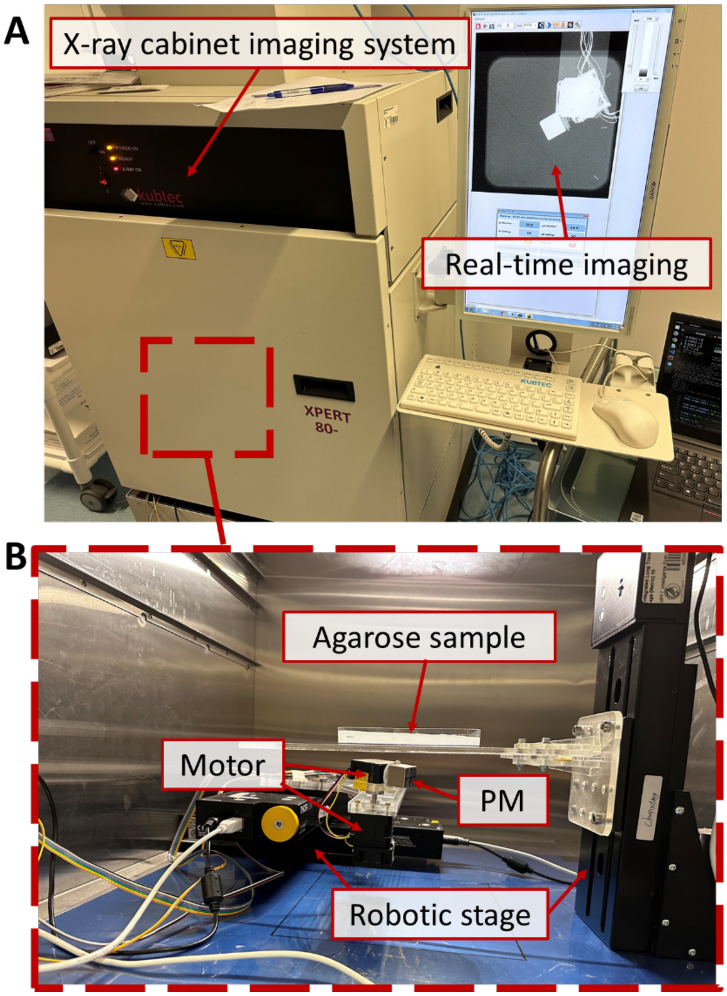

Fig. S19. Robotic system for multi-robot deployment under X-ray imaging. A. X-ray cabinet imaging system (XPERT® 80, KUBTEC® Scientific). **B.** 5-DOF robotic system for robot deployment inside the cabinet.

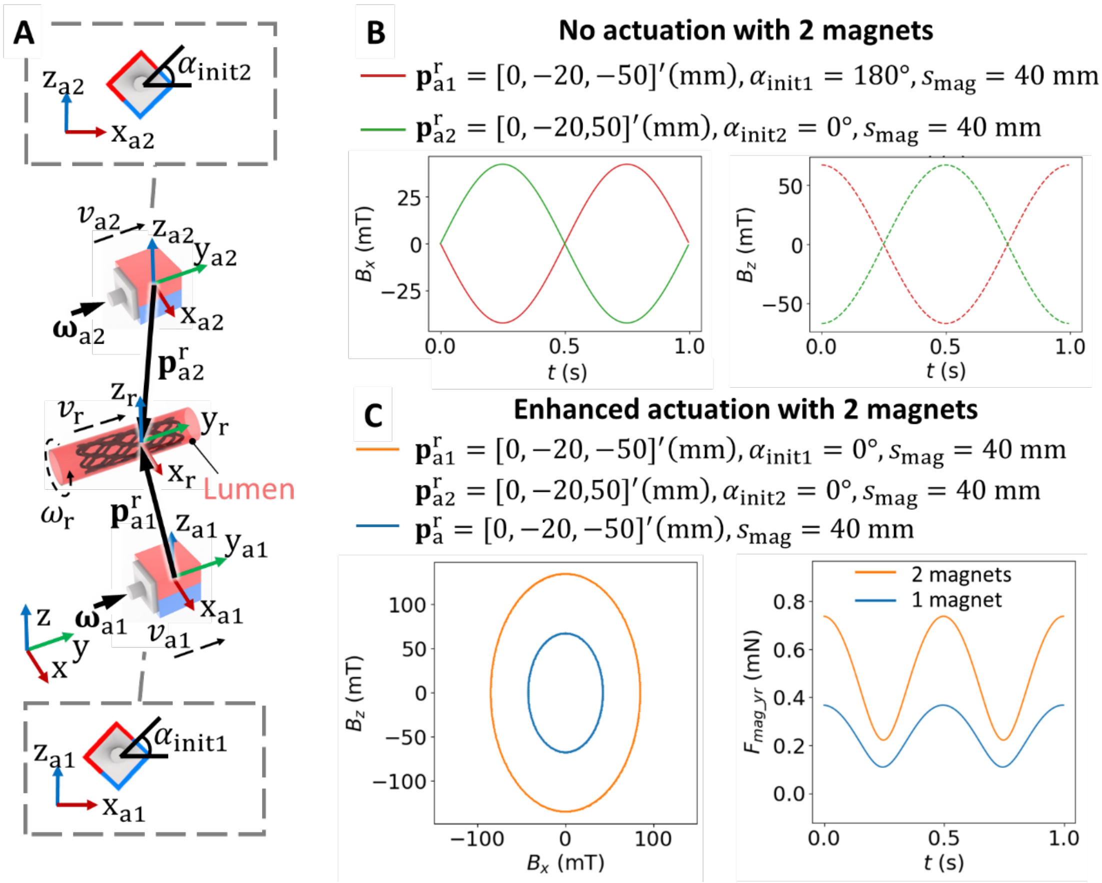

Fig. S20. Robot control with multiple permanent magnets. A. Single robot actuation with two permanent magnets. The variables for robot control include: magnet size (s_{mag}), relative magnet position ($\mathbf{p}_{\mathrm{a}}^{\mathrm{r}}$), rotational speed ($\boldsymbol{\omega}_{\mathrm{a}}$), and initial rotation angle (α_{init}). **B.** Neutralization of magnetic fields. The robot is unaffected by magnetic fields when the magnetic fields neutralize each other, such as two 40-mm magnets at $\mathbf{p}_{\mathrm{a1}}^{\mathrm{r}} = [0, -20\ \mathrm{mm}, 50\ \mathrm{mm}]'$ and $\mathbf{p}_{\mathrm{a2}}^{\mathrm{r}} = [0, -20\ \mathrm{mm}, -50\ \mathrm{mm}]'$ with $\alpha_{\mathrm{init1}} = 180°$ and $\alpha_{\mathrm{init2}} = 0°$, respectively. **C.** Enhanced actuation with two magnets. The amplitude of the magnetic field on the $\mathrm{x_r} - \mathrm{z_r}$ plane and the magnetic force along the y_{r}-axis ($F_{\mathrm{mag\_yr}}$) nearly double with two magnets compared with that of one magnet, when placing two 40-mm magnets at $\mathbf{p}_{\mathrm{a1}}^{\mathrm{r}} = [0, -20\ \mathrm{mm}, 50\ \mathrm{mm}]'$ and $\mathbf{p}_{\mathrm{a2}}^{\mathrm{r}} = [0, -20\ \mathrm{mm}, -50\ \mathrm{mm}]'$ with $\alpha_{\mathrm{init1}} = 0°$ and $\alpha_{\mathrm{init2}} = 0°$, respectively.

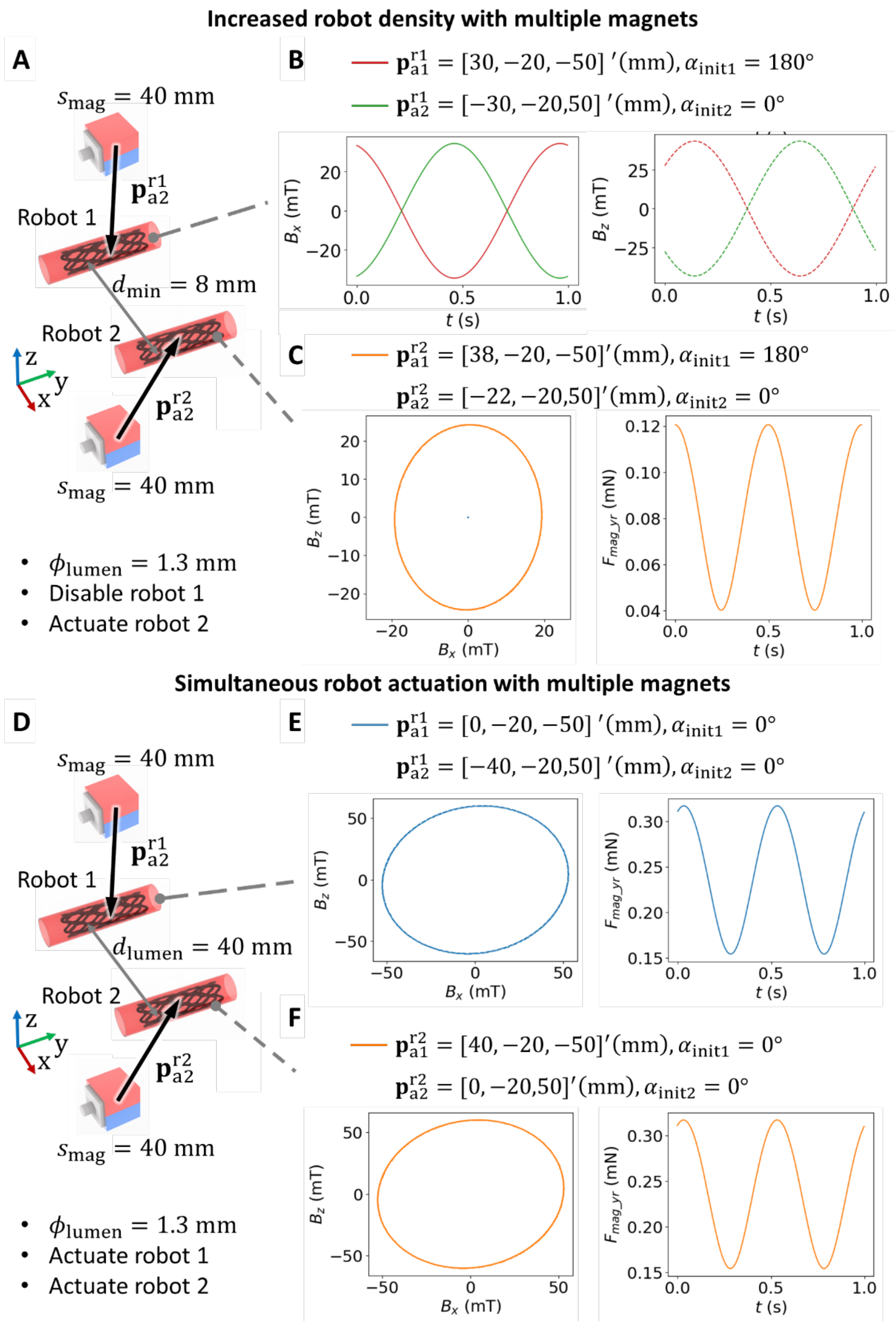

Fig. S21. Multiple robotic control with multiple permanent magnets. A. Independent control of two robots with two 40-mm permanent magnets inside straight 1.0 wt% agarose lumens of the diameter 1.3 mm. Independent control can be achieved by placing two permanent magnets at

$\mathbf{p}_{\mathrm{a1}}^{\mathrm{r1}} = [\Delta x, \Delta y, \Delta z]'$, $\mathbf{p}_{\mathrm{a2}}^{\mathrm{r1}} = [-\Delta x, \Delta y, -\Delta z]'$and $\mathbf{p}_{\mathrm{a1}}^{\mathrm{r2}} = [\Delta x + l_{\mathrm{min}}, \Delta y, \Delta z]'$, $\mathbf{p}_{\mathrm{a2}}^{\mathrm{r2}} = [-\Delta x + l_{\mathrm{min}}, \Delta y, -\Delta z]'$ with $\alpha_{\mathrm{init1}} = 180°$ and $\alpha_{\mathrm{init2}} = 0°$, respectively. The minimum robot distance d_{min} can be reduced to 8 mm compared with 67 mm with a single 40-mm permanent magnet. **B.** Neutralization of magnetic fields on robot 1. The rotation of robot 1 is disabled when two magnetic fields neutralize by placing two 40-mm magnets at $\mathbf{p}_{\mathrm{a1}}^{\mathrm{r1}} = [30\ \mathrm{mm}, -20\ \mathrm{mm}, 50\ \mathrm{mm}]'$ and $\mathbf{p}_{\mathrm{a2}}^{\mathrm{r1}} = [-30\ \mathrm{mm}, -20\ \mathrm{mm}, -50\ \mathrm{mm}]'$ with $\alpha_{\mathrm{init1}} = 180°$ and $\alpha_{\mathrm{init2}} = 0°$, respectively. **C.** Actuation of robot 2. Robot 2 can be actuated with two magnets at $\mathbf{p}_{\mathrm{a1}}^{\mathrm{r1}} = [38\ \mathrm{mm}, -20\ \mathrm{mm}, 50\ \mathrm{mm}]'$ and $\mathbf{p}_{\mathrm{a2}}^{\mathrm{r1}} = [-22\ \mathrm{mm}, -20\ \mathrm{mm}, -50\ \mathrm{mm}]'$ with $\alpha_{\mathrm{init1}} = 180°$ and $\alpha_{\mathrm{init2}} = 0°$, respectively. **D**. Simultaneous control of two robots with two 40-mm permanent magnets inside straight 1.0 wt% agarose lumens of the diameter 1.3 mm at the lumen distance $d_{\mathrm{lumen}} = 40$ mm. **E-F**. Magnetic fields and forces on robot 1 and 2. The magnets are placed at $\mathbf{p}_{\mathrm{a1}}^{\mathrm{r1}} = [0, -20\ \mathrm{mm}, -50\ \mathrm{mm}]'$, $\mathbf{p}_{\mathrm{a2}}^{\mathrm{r1}} = [-40\ \mathrm{mm}, -20\ \mathrm{mm}, 50\ \mathrm{mm}]'$and $\mathbf{p}_{\mathrm{a1}}^{\mathrm{r2}} = [40\ \mathrm{mm}, -20\ \mathrm{mm}, -50\ \mathrm{mm}]'$, $\mathbf{p}_{\mathrm{a2}}^{\mathrm{r2}} = [0, -20\ \mathrm{mm}, -50\ \mathrm{mm}]'$ with $\alpha_{\mathrm{init1}} = 0°$ and $\alpha_{\mathrm{init2}} = 0°$, respectively.

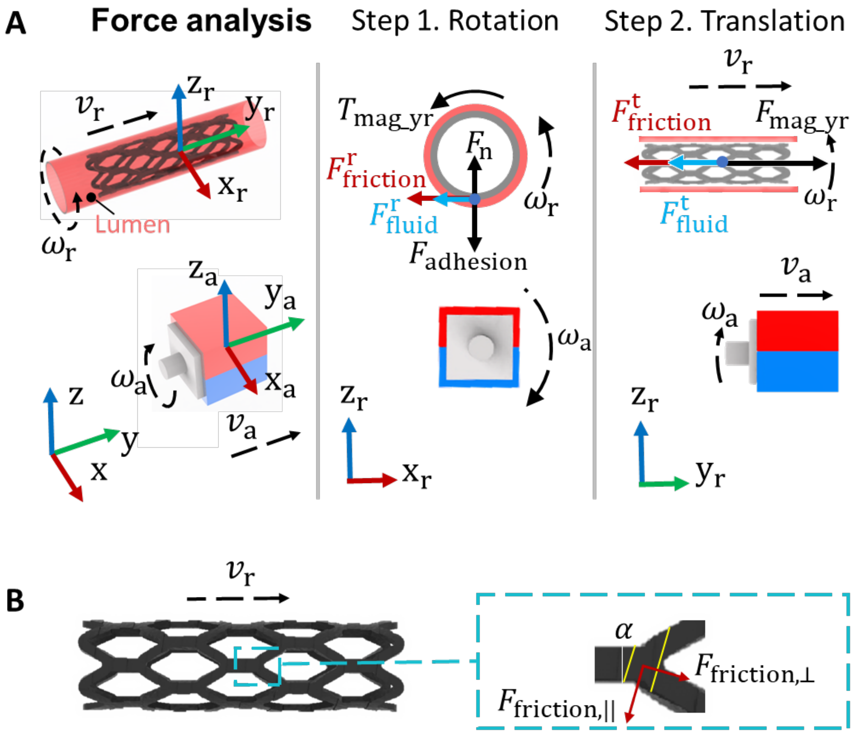

Fig. S22. Force analysis of the robot locomotion. A. Force analysis of the robot. **B.** Friction on the robot.

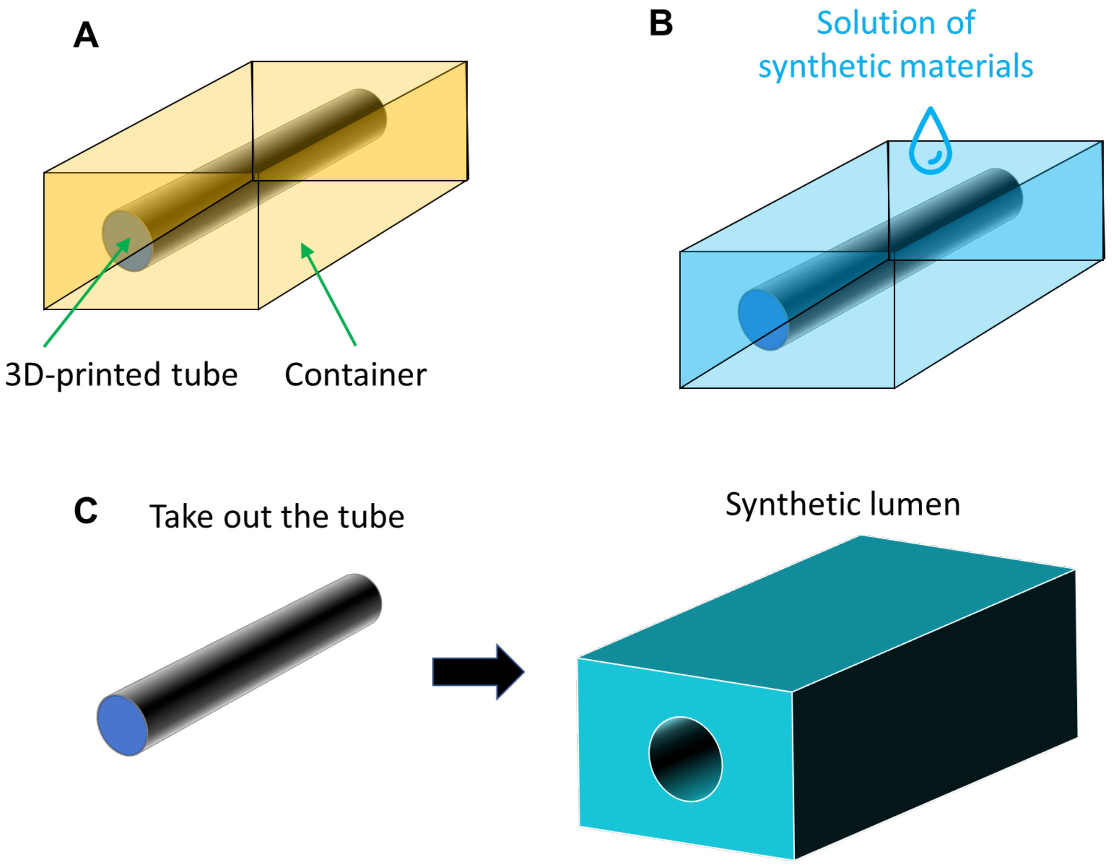

Fig. S23. Preparation of synthetic phantoms. A. 3d-printed tube as the positive mold. **B.** Solution of synthetic materials poured into the container. **C.** Fabricated lumen by extracting the tube from cured synthetic material.

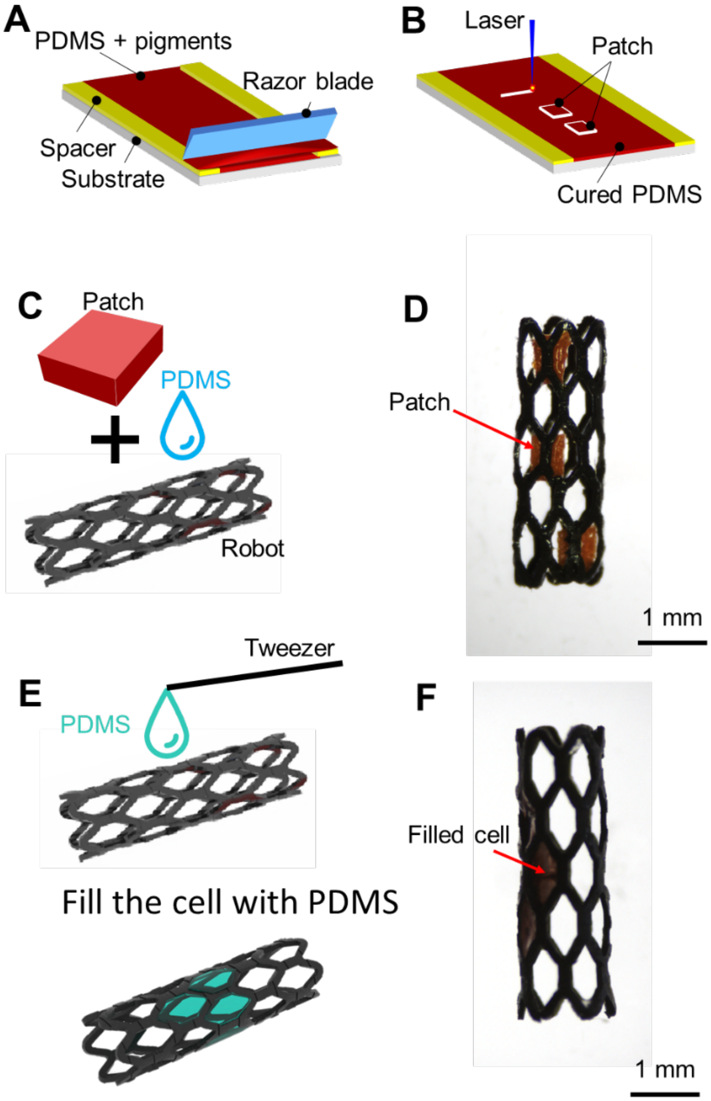

Fig. S24. Preparation of the proof-of-concept functional modules. A. Fabrication of the PDMS sheet. The mixture of pigments and PDMS was poured onto a PMMA substrate, after which a razor blade scratched against the spacer for a uniform sheet thickness. **B.** Fabrication of the patch. The PDMS sheet was cut into the rectangular segment with specified dimensions using a laser machine (LPKF ProtoLaser U3, LPKF Laser & Electronics AG). **C.** Integration of the patch into the robot with PDMS. **D**. Fabricated robot with PDMS patches. **E.** Fabrication of the flow diverters by sealing certain stent cells with PDMS. **F.** Fabricated robot as an active flow diverter.

Supplementary Tables

Table S1. Benefits of using multiple robots for multi-site diseases in distal cortical artery segments.

**Medical procedures**	**Multi-site disease occurrence rate**	**Benefits of using multiple robots**
Thrombolysis for acute ischemic stroke	16 % for ACA/M3/M4 (*70*);<br><br>24%-40% for distal, medium vessel occlusions (*40*);<br><br>The frequency of multiple ischemic lesions was approximately 50% (*71*).	1. Spatial efficiency: on-site *drug delivery* directly to vicinity of multi-site strokes, reducing the systematic dosage while maintaining the efficacy.<br><br>2. Temporal efficiency: enhanced navigation and *drug delivery* efficiency for a prompter treatment of multi-site strokes in distal regions compared with a single robot or catherization.<br><br>3. Improved efficiency for *heterogenous drug delivery*, i.e., the *requirement* of different drugs targeting different locations, to different lesions.
Embolization and flow diversion for aneurysms	Approximately 10% to 30% of people who have a brain aneurysm have multiple aneurysms (*72*).	1. Fulfilling the *requirement* of *drug delivery*, *embolization* and *flow diversion* for treatment of multi-site diseases.<br><br>2. Enhanced spatial and temporal efficiency for multi-site heterogeneous *drug delivery* and *embolization* for treatment and prompt management of potential post-treatment risks.<br><br>3. Improved multi-site *flow diversion* efficiency and
Embolization for cerebral arterial-venous malformation (AVM)	6 out of 7 at cortical region (*73*);<br><br>Around 9% of patients could be included in the multiple arteriovenous malformation category (*74, 75*).	
Embolization for dural arteriovenous fistulas (DVAFs)	Multiple DAVFs at separate sites are relatively rare, accounting for 7% of all intracranial DAVFs (*76*).	
Therapeutic agents delivery for brain tumors	Most malignant primary brain tumors occur in the cerebral cortex, with the highest percentage 26% developing in the frontal lobe (*77*).	

	Volume of tumors can range from 27 mm^3 to 23000 mm^3 (*78*).	adjustability of multi-site flow diverters after full deployment.

Supplementary Movies

Movie S1. Multi-robot deployment within a 3D lumen network.
This video shows the deployment and retrieval of four robots within a single-layer 3D lumen network with a 50 mm cubic permanent magnet. The blood analog with 48 ml/min with a stroke rate of 80 bpm was pumped into the phantom during the experiment, which was also visualized in the video.

Movie S2. Multi-robot deployment within a multi-layer lumen network.
This video shows the deployment and retrieval of six robots within a double-layer 3D lumen network. A 50 mm cubic permanent magnet was utilized to actuate robots on the lower layer, while a 40 mm cubic permanent magnet was employed for robots on the upper layer. The blood analog with 48 ml/min with a stroke rate of 80 bpm was pumped into the phantom during the experiment**.**

Movie S3. Multi-robot deployment under real-time medical imaging.
This video demonstrates the deployment and retrieval of four robots within a double-layer agarose gel phantom under X-ray cabinet imaging, and the sequential deployment of three robots into the porcine artery, a lumen of agarose gel, and a lumen of PDMS. The robot deployment inside the porcine artery was monitored by the ultrasound imaging, and phosphate-buffered saline was pumped to the artery at a flow rate of 10–12 ml/min.

Movie S4. Simultaneous multi-site agent delivery with multiple robots.
This video shows the deployment of three robots with functional patches (PDMS patches containing embedded pigments) to the target locations and the gradual release of various pigments into the surrounding area over 24 hours at multiple locations simultaneously.

Movie S5. Simultaneous multi-site flow diversion with multiple robots.
This video shows the deployment of three robots with diversion patches to obstruct the fluid flow to branch lumens. Water was pumped into the lumen at a flow rate of 48 ml/min with a stroke rate of 80 bpm, during which the dyed water was injected to visualize the diversion effect.

REFERENCES AND NOTES

1. M. Sitti, *Mobile microrobotics* (MIT Press, Cambridge, MA, 2017).
2. M. Cianchetti, C. Laschi, A. Menciassi, P. Dario, Biomedical applications of soft robotics. *Nat. Rev. Mater.* **3**, 143–153 (2018).
3. T. Wang, Y. Wu, E. Yildiz, S. Kanyas, M. Sitti, Clinical translation of wireless soft robotic medical devices. *Nat. Rev. Bioeng.* **2**, 470–485 (2024).
4. M. Z. Miskin, A. J. Cortese, K. Dorsey, E. P. Esposito, M. F. Reynolds, Q. Liu, M. Cao, D. A. Muller, P. L. McEuen, I. Cohen, Electronically integrated, mass-manufactured, microscopic robots. *Nature* **584**, 557–561 (2020).
5. S. Palagi, A. G. Mark, S. Y. Reigh, K. Melde, T. Qiu, H. Zeng, C. Parmeggiani, D. Martella, A. Sanchez-Castillo, N. Kapernaum, Structured light enables biomimetic swimming and versatile locomotion of photoresponsive soft microrobots. *Nat. Mater.* **15**, 647–653 (2016).
6. M. D. Brown, B. T. Cox, B. E. Treeby, Stackable acoustic holograms. *Appl. Phys. Lett.* **116**, 261901 (2020).
7. Z. Ma, K. Melde, A. G. Athanassiadis, M. Schau, H. Richter, T. Qiu, P. Fischer, Spatial ultrasound modulation by digitally controlling microbubble arrays. *Nat. Commun.* **11**, 4537 (2020).
8. B. Hao, X. Wang, Y. Dong, M. Sun, C. Xin, H. Yang, Y. Cao, J. Zhu, X. Liu, C. Zhang, Focused ultrasound enables selective actuation and Newton-level force output of untethered soft robots. *Nat. Commun.* **15**, 5197 (2024).
9. W. Hu, G. Z. Lum, M. Mastrangeli, M. Sitti, Small-scale soft-bodied robot with multimodal locomotion. *Nature* **554**, 81–85 (2018).
10. T. Wang, H. Ugurlu, Y. Yan, M. Li, M. Li, A.-M. Wild, E. Yildiz, M. Schneider, D. Sheehan, W. Hu, Adaptive wireless millirobotic locomotion into distal vasculature. *Nat. Commun.* **13**, 4465 (2022).

11. M. Sitti, Miniature soft robots—Road to the clinic. *Nat. Rev. Mater.* **3**, 74–75 (2018).

12. M. Sitti, H. Ceylan, W. Hu, J. Giltinan, M. Turan, S. Yim, E. Diller, Biomedical applications of untethered mobile milli/microrobots. *Proc. IEEE Inst. Electr. Electron Eng.* **103**, 205–224 (2015).

13. M. Li, A. Pal, A. Aghakhani, A. Pena-Francesch, M. Sitti, Soft actuators for real-world applications. *Nat. Rev. Mater.* **7**, 235–249 (2022).

14. S. Lee, S. Kim, S. Kim, J. Y. Kim, C. Moon, B. J. Nelson, H. Choi, A capsule-type microrobot with pick-and-drop motion for targeted drug and cell delivery. *Adv. Healthc. Mater.* **7**, e1700985 (2018).

15. J. Tang, C. Yao, Z. Gu, S. Jung, D. Luo, D. Yang, Super-soft and super-elastic DNA robot with magnetically driven navigational locomotion for cell delivery in confined space. *Angew. Chem. Int. Ed. Engl.* **59**, 2490–2495 (2020).

16. B. Wang, K. F. Chan, K. Yuan, Q. Wang, X. Xia, L. Yang, H. Ko, Y.-X. J. Wang, J. J. Y. Sung, P. W. Y. Chiu, Endoscopy-assisted magnetic navigation of biohybrid soft microrobots with rapid endoluminal delivery and imaging. *Sci. Robot.* **6**, eabd2813 (2021).

17. Y. Kim, E. Genevriere, P. Harker, J. Choe, M. Balicki, R. W. Regenhardt, J. E. Vranic, A. A. Dmytriw, A. B. Patel, X. Zhao, Telerobotic neurovascular interventions with magnetic manipulation. *Sci. Robot.* **7**, eabg9907 (2022).

18. X. Liu, L. Wang, Y. Xiang, F. Liao, N. Li, J. Li, J. Wang, Q. Wu, C. Zhou, Y. Yang, Magnetic soft microfiberbots for robotic embolization. *Sci. Robot.* **9**, eadh2479 (2024).

19. J. Law, X. Wang, M. Luo, L. Xin, X. Du, W. Dou, T. Wang, G. Shan, Y. Wang, P. Song, Microrobotic swarms for selective embolization. *Sci. Adv.* **8**, eabm5752 (2022).

20. R. H. Soon, Z. Yin, M. A. Dogan, N. O. Dogan, M. E. Tiryaki, A. C. Karacakol, A. Aydin, P. Esmaeili-Dokht, M. Sitti, Pangolin-inspired untethered magnetic robot for on-demand biomedical heating applications. *Nat. Commun.* **14**, 3320 (2023).

21. C. Wang, Y. Wu, X. Dong, M. Armacki, M. Sitti, In situ sensing physiological properties of biological tissues using wireless miniature soft robots. *Sci. Adv.* **9**, eadg3988 (2023).

22. J. Han, X. Dong, Z. Yin, S. Zhang, M. Li, Z. Zheng, M. C. Ugurlu, W. Jiang, H. Liu, M. Sitti, Actuation-enhanced multifunctional sensing and information recognition by magnetic artificial cilia arrays. *Proc. Natl. Acad. Sci. U.S.A.* **120**, e2308301120 (2023).

23. B. Xiao, Y. Xu, S. Edwards, L. Balakumar, X. Dong, Sensing mucus physiological property in situ by wireless millimeter-scale soft robots. *Adv. Funct. Mater.* **34**, 2307751 (2024).

24. J. J. Abbott, E. Diller, A. J. Petruska, Magnetic methods in robotics. *Annu. Rev. Control Robot. Auton. Syst.* **3**, 57–90 (2020).

25. Y. Wu, X. Dong, J.-k. Kim, C. Wang, M. Sitti, Wireless soft millirobots for climbing three-dimensional surfaces in confined spaces. *Sci. Adv.* **8**, eabn3431 (2022).

26. J. Zhang, Z. Ren, W. Hu, R. H. Soon, I. C. Yasa, Z. Liu, M. Sitti, Voxelated three-dimensional miniature magnetic soft machines via multimaterial heterogeneous assembly. *Sci. Robot.* **6**, eabf0112 (2021).

27. Z. Ren, R. Zhang, R. H. Soon, Z. Liu, W. Hu, P. R. Onck, M. Sitti, Soft-bodied adaptive multimodal locomotion strategies in fluid-filled confined spaces. *Sci. Adv.* **7**, eabh2022 (2021).

28. J. R. McFaline-Figueroa, E. Q. Lee, Brain tumors. *Am. J. Med.* **131**, 874–882 (2018).

29. T. Tunthanathip, K. Kanjanapradit, S. Ratanalert, N. Phuenpathom, T. Oearsakul, A. Kaewborisutsakul, Multiple, primary brain tumors with diverse origins and different localizations: Case series and review of the literature. *J. Neurosci. Rural Pract.* **9**, 593–607 (2018).

30. M. Nasor, W. Obaid, Detection and localization of early-stage multiple brain tumors using a hybrid technique of patch-based processing, k-means clustering and object counting. *Int. J. Biomed. Imaging* **2020**, 9035096 (**2020**).

31. K. Ikeda, H. Wakimoto, T. Ichikawa, S. Jhung, F. H. Hochberg, D. N. Louis, E. A. Chiocca, Complement depletion facilitates the infection of multiple brain tumors by an intravascular, replication-conditional herpes simplex virus mutant. *J. Virol.* **74**, 4765–4775 (2000).

32. J. Rahmer, C. Stehning, B. Gleich, Spatially selective remote magnetic actuation of identical helical micromachines. *Sci. Robot.* **2**, eaal2845 (2017).

33. S. Juvela, Risk factors for multiple intracranial aneurysms. *Stroke* **31**, 392–397 (2000).

34. K. Mizoi, J. Suzuki, T. Yoshimoto, Surgical treatment of multiple aneurysms: Review of experience with 372 cases. *Acta Neurochir.* **96**, 8–14 (1989).

35. J. Hu, H. Albadawi, B. W. Chong, A. R. Deipolyi, R. A. Sheth, A. Khademhosseini, R. Oklu, Advances in biomaterials and technologies for vascular embolization. *Adv. Mater.* **31**, e1901071 (2019).

36. D.-H. Kim, N. Lu, R. Ghaffari, Y.-S. Kim, S. P. Lee, L. Xu, J. Wu, R.-H. Kim, J. Song, Z. Liu, Materials for multifunctional balloon catheters with capabilities in cardiac electrophysiological mapping and ablation therapy. *Nat. Mater.* **10**, 316–323 (2011).

37. D.-H. Kim, R. Ghaffari, N. Lu, S. Wang, S. P. Lee, H. Keum, R. D'Angelo, L. Klinker, Y. Su, C. Lu, Electronic sensor and actuator webs for large-area complex geometry cardiac mapping and therapy. *Proc. Natl. Acad. Sci. U.S.A.* **109**, 19910–19915 (2012).

38. M. Han, L. Chen, K. Aras, C. Liang, X. Chen, H. Zhao, K. Li, N. R. Faye, B. Sun, J.-H. Kim, Catheter-integrated soft multilayer electronic arrays for multiplexed sensing and actuation during cardiac surgery. *Nat. Biomed. Eng.* **4**, 997–1009 (2020).

39. B. H. Kim, K. Li, J.-T. Kim, Y. Park, H. Jang, X. Wang, Z. Xie, S. M. Won, H.-J. Yoon, G. Lee, Three-dimensional electronic microfliers inspired by wind-dispersed seeds. *Nature* **597**, 503–510 (2021).

40. J. L. Saver, R. Chapot, R. Agid, A. E. Hassan, A. P. Jadhav, D. S. Liebeskind, K. Lobotesis, D. Meila, L. Meyer, G. Raphaeli, Thrombectomy for distal, medium vessel occlusions: A consensus statement on present knowledge and promising directions. *Stroke* **51**, 2872–2884 (2020).

41. A. R. Xavier, A. M. Siddiqui, J. F. Kirmani, R. A. Hanel, A. M. Yahia, A. I. Qureshi, Clinical potential of intra-arterial thrombolytic therapy in patients with acute ischaemic stroke. *CNS Drugs* **17**, 213–224 (2003).

42. J. A. Grossberg, L. C. Rebello, D. C. Haussen, M. Bouslama, M. Bowen, C. M. Barreira, S. R. Belagaje, M. R. Frankel, R. G. Nogueira, Beyond large vessel occlusion strokes: Distal occlusion thrombectomy. *Stroke* **49**, 1662–1668 (2018).

43. Y. Kantaros, B. V. Johnson, S. Chowdhury, D. J. Cappelleri, M. M. Zavlanos, Control of magnetic microrobot teams for temporal micromanipulation tasks. *IEEE Trans. Robot.* **34**, 1472–1489 (2018).

44. B. V. Johnson, S. Chowdhury, D. J. Cappelleri, Local magnetic field design and characterization for independent closed-loop control of multiple mobile microrobots. *IEEE/ASME Trans. Mechatron.* **25**, 526–534 (2020).

45. E. Diller, C. Pawashe, S. Floyd, M. Sitti, Assembly and disassembly of magnetic mobile micro-robots towards deterministic 2-D reconfigurable micro-systems. *Int. J. Robot. Res.* **30**, 1667–1680 (2011).

46. X. Fan, X. Dong, A. C. Karacakol, H. Xie, M. Sitti, Reconfigurable multifunctional ferrofluid droplet robots. *Proc. Natl. Acad. Sci. U.S.A.* **117**, 27916–27926 (2020).

47. X. Dong, M. Sitti, Controlling two-dimensional collective formation and cooperative behavior of magnetic microrobot swarms. *Int. J. Robot. Res.* **39**, 617–638 (2020).

48. L. Yang, J. Jiang, X. Gao, Q. Wang, Q. Dou, L. Zhang, Autonomous environment-adaptive microrobot swarm navigation enabled by deep learning-based real-time distribution planning. *Nat. Mach. Intell.* **4**, 480–493 (2022).

49. F. Ongaro, S. Pane, S. Scheggi, S. Misra, Design of an electromagnetic setup for independent three-dimensional control of pairs of identical and nonidentical microrobots. *IEEE Trans. Robot.* **35**, 174–183 (2019).

50. S. Shahrokhi, J. Shi, B. Isichei, A. T. Becker, Exploiting nonslip wall contacts to position two particles using the same control input. *IEEE Trans. Robot.* **35**, 577–588 (2019).

51. T. Xu, C. Huang, Z. Lai, X. Wu, Independent control strategy of multiple magnetic flexible millirobots for position control and path following. *IEEE Trans. Robot.* **38**, 2875–2887 (2022).

52. E. Diller, J. Giltinan, M. Sitti, Independent control of multiple magnetic microrobots in three dimensions. *Int. J. Robot. Res.* **32**, 614–631 (2013).

53. S. Floyd, E. Diller, C. Pawashe, M. Sitti, Control methodologies for a heterogeneous group of untethered magnetic micro-robots. *Int. J. Robot. Res.* **30**, 1553–1565 (2011).

54. E. Diller, S. Floyd, C. Pawashe, M. Sitti, Control of multiple heterogeneous magnetic microrobots in two dimensions on nonspecialized surfaces. *IEEE Trans. Robot.* **28**, 172–182 (2012).

55. L. Amoudruz, P. Koumoutsakos, Independent control and path planning of microswimmers with a uniform magnetic field. *Adv. Intell. Syst.* **4**, 2100183 (2022).

56. L. Yang, L. Zhang, Motion control in magnetic microrobotics: From individual and multiple robots to swarms. *Ann. Rev. Control Robot. Auton. Syst.* **4**, 509–534 (2021).

57. M. Salehizadeh, E. D. Diller, Path planning and tracking for an underactuated two-microrobot system. *IEEE Robot. Autom. Lett.* **6**, 2674–2681 (2021).

58. A. Denasi, S. Misra, Independent and leader–follower control for two magnetic micro-agents. *IEEE Robot. Autom. Lett.* **3**, 218–225 (2018).

59. J. Davy, T. Da Veiga, G. Pittiglio, J. H. Chandler, P. Valdastri, in *2023 International Symposium on Medical Robotics (ISMR)*. (IEEE, 2023), pp. 1–7.

60. Z. Koszowska, M. Brockdorff, T. da Veiga, G. Pittiglio, P. Lloyd, T. Khan-White, R. A. Harris, J. W. Moor, J. H. Chandler, P. Valdastri, Independently actuated soft magnetic manipulators for bimanual operations in confined anatomical cavities. *Adv. Intell. Syst.* **6**, 2300062 (2024).

61. P. Ryan, E. Diller, in *2016 IEEE international conference on robotics and automation (ICRA).* (IEEE, 2016), pp. 1731–1736.

62. A. W. Mahoney, J. J. Abbott, Generating rotating magnetic fields with a single permanent magnet for propulsion of untethered magnetic devices in a lumen. *IEEE Trans. Robot.* **30**, 411–420 (2014).

63. P. E. Hart, N. J. Nilsson, B. Raphael, A formal basis for the heuristic determination of minimum cost paths. *IEEE Trans. Syst. Sci. Cybern.* **4**, 100–107 (1968).

64. M. F. Hale, R. Sidhu, M. E. McAlindon, Capsule endoscopy: Current practice and future directions. *World J. Gastroenterol.* **20**, 7752–7759 (2014).

65. C. McCaffrey, O. Chevalerias, C. O'Mathuna, K. Twomey, Swallowable-capsule technology. *IEEE Pervasive Comput* **7**, 23–29 (2008).

66. M. R. Bennett, J. Hasty, Microfluidic devices for measuring gene network dynamics in single cells. *Nat. Rev. Genet.* **10**, 628–638 (2009).

67. H. Song, J. D. Tice, R. F. Ismagilov, A microfluidic system for controlling reaction networks in time. *Angew. Chem. Int. Ed. Engl.* **115**, 792–796 (2003).

68. Y. Tang, M. Li, T. Wang, X. Dong, W. Hu, M. Sitti, Wireless miniature magnetic phase-change soft actuators. *Adv. Mater.* **34**, e2204185 (2022).

69. T. Chung, *Computational fluid dynamics* (Cambridge Univ. Press, 2010).

70. H. Zhao, S. Coote, L. Pesavento, L. Churilov, H. M. Dewey, S. M. Davis, B. C. Campbell, Large vessel occlusion scales increase delivery to endovascular centers without excessive harm from misclassifications. *Stroke* **48**, 568–573 (2017).

71. A. K. Bonkhoff, T. Ullberg, M. Bretzner, S. Hong, M. D. Schirmer, R. W. Regenhardt, K. L. Donahue, M. J. Nardin, A. V. Dalca, A.-K. Giese, Deep profiling of multiple ischemic lesions in a large, multi-center cohort: Frequency, spatial distribution, and associations to clinical characteristics. *Front. Neurosci.* **16**, 994458 (2022).

72. J. L. Brisman, J. K. Song, D. W. Newell, Cerebral aneurysms. *N. Engl. J. Med.* **355**, 928–939 (2006).

73. M. N. Shah, S. E. Smith, D. L. Dierker, J. P. Herbert, T. S. Coalson, B. S. Bruck, G. J. Zipfel, D. C. Van Essen, R. G. Dacey, The relationship of cortical folding and brain arteriovenous malformations. *Neurovasc. Imaging* **2**, 1–10 (2016).

74. R. Willinsky, P. Lasjaunias, K. Terbrugge, P. Burrows, Multiple cerebral arteriovenous malformations (AVMs) Review of our experience from 203 patients with cerebral vascular lesions. *Neuroradiology* **32**, 207–210 (1990).

75. K.-P. Stein, I. Wanke, N. Oezkan, Y. Zhu, I. E. Sandalcioglu, M. Forsting, U. Sure, Multiple cerebral arterio-venous malformations: Impact of multiplicity and hemodynamics on treatment strategies. *Acta Neurochir.* **158**, 2399–2407 (2016).

76. A. Rahmanian, M. R. Farrokhi, E. A. Alibai, M. S. Masoudi, Multiple intracranial dural arteriovenous fistula. *J. Res. Med. Sci.* **18**, 360–362 (2013).

77. J. Gould, A. McDonald, Breaking down brain cancer. *Nature* **561**, S40–S41 (2018).

78. M. F. Dempsey, B. R. Condon, D. M. Hadley, Measurement of tumor “size” in recurrent malignant glioma: 1D, 2D, or 3D? *AJNR Am. J. Neuroradiol.* **26**, 770–776 (2005).
